# Taxonomic and Functional Comparative Metagenomics of Peruvian Salterns: Insights into Microbial Communities and Aminotransferase Potential

**DOI:** 10.3390/microorganisms14071595

**Published:** 2026-07-22

**Authors:** Carol N. Flores-Fernández, Thomas K. Hiron, Dragana Dobrijevic, Amparo I. Zavaleta, Jack W. E. Jeffries, Chris A. O’Callaghan, Gary J. Lye, John M. Ward, Max Cárdenas-Fernández

**Affiliations:** 1 Centre for Human Genetics, Nuffield Department of Medicine, University of Oxford, Roosevelt Drive, Oxford OX3 7BN, UK; nathali.flores@well.ox.ac.uk (C.N.F.-F.); thomas.hiron@dpag.ox.ac.uk (T.K.H.); chris.ocallaghan@ndm.ox.ac.uk (C.A.O.); 2 Laboratorio de Biología Molecular, Facultad de Farmacia y Bioquímica, Universidad Nacional Mayor de San Marcos, Lima 15001, Peru; azavaletap@unmsm.edu.pe; 3 Department of Biochemical Engineering, The Advanced Centre for Biochemical Engineering, University College London, London WC1E 6BT, UK; draganadobrijevic@gmail.com (D.D.); g.lye@ucl.ac.uk (G.J.L.); 4 Department of Biochemical Engineering, University College London, Bernard Katz Building, Gower Street, London WC1E 6BT, UK; jack.jeffries.12@ucl.ac.uk; 5 Energy and Bioproducts Research Institute, Aston University, Birmingham B4 7ET, UK; 6 Department of Chemical and Bioprocess Engineering, School of Engineering and Innovation, Aston University, Birmingham B4 7ET, UK

**Keywords:** extreme environments, extremophiles, halophiles, microbial diversity, metabolic pathways, metagenome-assembled genomes, aminotransferases class III

## Abstract

Metagenomic analysis of extreme environments is essential in biotechnological research. This work aimed to determine and compare the microbial diversity of two Peruvian saline environments and characterise their functional profiles. Soil metagenomic DNA (mDNA) was analysed from Maras and Pilluana salterns, both with a thalassohaline origin but with different geographical and environmental conditions. Maras samples exhibited more diversity and a remarkably higher abundance of archaea (phylum *Euryarchaeota*). The most dominant bacterial phyla across all the samples were *Pseudomonadota* and *Actinomycetota*. Multiple pathways specific to archaea were more abundant in Maras, as were pathway-related synthesis and degradation of compatible osmolytes such as glycine betaine and ectoine. The most abundant pathways in Pilluana were associated with fatty acid biosynthesis and oxidation. A total of 49 and 47 metagenomic-assembled genomes (MAGs) were retrieved from Maras and Pilluana samples, respectively. Bacterial MAGs were mainly classified within the phyla *Psudomonadota*, *Actinomycetota*, *Planctomycetota*, and *Gemmatimonadota*. Additionally, a total of 20 putative aminotransferases class III (ATs, PF00202) from Maras3 were cloned and expressed in *E. coli* Rosetta, and their substrate scoping was assayed against several aldehyde and ketone substrates; AT pQR3082 and pQR3090 showed unique broad substrate acceptance for aromatic and aliphatic substrates. Our study provides new insights into the microorganisms and metabolic pathways of these unique extreme environments, highlighting the promising biotechnological potential of metagenomic ATs.

## 1. Introduction

Microorganisms and their genetic information are critical in industrial biotechnology and sustainable growth, as they are sources of bioactive compounds such as enzymes, peptides, antimicrobials, and biosurfactants [1,2]. Microbial communities have mainly been studied through culture-dependent and 16S rRNA gene analysis methods [3]. The former enables the isolation of less than 1% of microbial diversity in pure culture, whilst the latter does not provide deep taxonomic information or give access to any enzyme encoding gene sequences. In addition, neither of these methods can provide functional profiles nor the discovery of novel biomolecules from uncultivable microbes [4]. Genome mining of cultivable strains is used to find novel biomolecules such as enzymes and secondary metabolites; however, the very low percentage of cultivable strains limits its wider discovery potential [5].

In contrast, metagenomic techniques overcome culture-dependent limitations and represent a valuable tool for studying the microbial biodiversity of complex environments, identifying uncultivable microorganisms and discovering novel biomolecules [6,7]. There are two main metagenomic approaches: structural metagenomics, which studies microbial populations and their dynamics under the specific conditions of an ecosystem; and functional metagenomics, which identifies genes that encode a function of interest. The analysis of 16S rRNA genes is linked with both metagenomic approaches [8]. Over the last decade, metagenomics empowered by next-generation sequencing (NGS) has enabled the determination of taxonomic and functional profiles of given samples in a specific environment [3,9,10].

Saline environments are characterised by factors that can restrict microbial life such as high salt concentration, low oxygen content, low or high temperatures, sometimes alkalinity, and the presence of toxic compounds [6]. Thus, these environments represent valuable niches for studying the adaptability mechanisms and biotechnological potential of unique microbial communities that have adapted and thrived under such extreme conditions [11]. Halophile microorganisms grow in these extreme saline environments, developing potential for biotechnological applications in bioremediation, as well as in the production of biopharmaceuticals, biochemicals and enzymes for industrial use [4,12,13].

Metagenomics has been applied to study hypersaline environments around the world to reveal their microbial diversity. Metagenomic studies have focused on describing the taxonomic and functional profiles of the microbiota, their interactions with the environment, and their relationship with the evolutive process. Furthermore, they have provided insights into the biotechnological potential of the indigenous microbiota [4,6,10,11,12,14]. Peruvian salterns are unique environments, and there is very limited information regarding their microbial diversity, taxonomy and functional profiles. Maturrano et al. (2006) determined the microbiota in water samples from Maras salterns by fluorescence in situ hybridisation (FISH), 16S rRNA gene clone library analysis, and cultivation techniques [15]. Castelán-Sánchez et al. (2019) also analysed water samples from Maras salterns using a metagenomic approach to conduct a taxonomic analysis [16]. To the best of our knowledge, taxonomic and functional metagenomic studies have not been carried out in conjunction in Peruvian salterns to date.

This work aimed to determine and compare the microbial and functional diversity from two Peruvian saline environments using a metagenomic approach and analyse potential for biotechnological application with focus on industrially relevant aminotransferases. To achieve this, soil metagenomic DNAs (mDNAs) from Maras and Pilluana Peruvian salterns were extracted and fully sequenced by NGS, and then their taxonomic and functional profiles were determined. The metagenome-assembled genomes (MAGs) were retrieved and their genetic information was analysed. In addition, aminotransferases class III were identified and cloned and their bioprospecting toward several substrates was determined. The results we report expand knowledge of the indigenous microbial communities in these environments and provide further insights into their biotechnological potential.

## 2. Materials and Methods

### 2.1. Site of Study and Sample Collection

**Figure 1:**
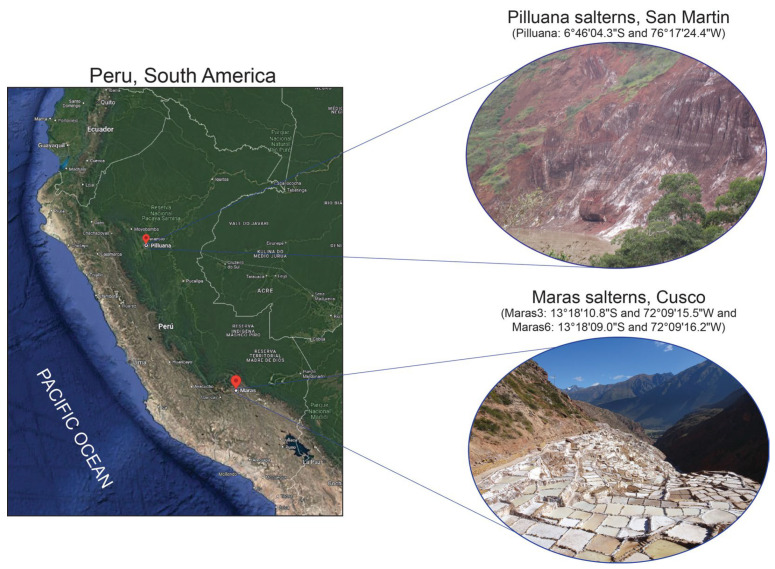
Geographical location of Pilluana and Maras salterns in Peru.

Two soil samples were collected from Maras salterns (Cusco, Peru), coded as Maras3 (13°18′10.8″ S and 72°09′15.5″ W) and Maras6 (13°18′09.0″ S and 72°09′16.2″ W). One soil sample was collected from Pilluana salterns (San Martín, Perú), coded as Pilluana (6°46′04.3″ S and 76°17′24.4″ W) (Figure 1). The Maras3 site had friable red soil near some plants just above running saline water. Maras6 site also had friable red soil, and it was a dug-over pond where the salt had been removed and the soil had been dug over but not yet smoothed to revitalise it. The two sampling sites were approximately 90 m apart. The parameters of the sample sites are presented in Supplementary Table S1. The soil samples were collected from a few centimetres below the surface, kept in sterile plastic containers and maintained at 4 °C.

### 2.2. mDNA Extraction

For mDNA extraction, pre-lysis washing was performed by mixing 10 g of soil sample with 15 mL of washing buffer (100 mM Tris, 300 mM NaCl, 100 mM EDTA, 100 mM NaH_2_PO_4_ and 100 mM Na_2_HPO_4_, pH 8) in a water bath with orbital agitation at 160 rpm and 65 °C for 20 min. After centrifugation (12,000 *g* at 4 °C for 10 min), the pellet was recovered and the procedure was repeated four times. The final pellet was re-suspended in 12.5 mL of washing buffer containing 1.5 mg/mL of lysozyme and incubated for 1 h at 37 °C. To this mixture, 215 μL of proteinase K (10 mg/mL) was added and it was incubated for 1 h at 65 °C. Then, 650 μL of SDS (20%) was added and it was incubated at 160 rpm and 65 °C for 2 h. The supernatant was recovered by centrifugation at 12,000 *g* for 10 min and mixed with an equal volume of phenol, chloroform, and isoamyl alcohol in a ratio of 25:24:1. The aqueous phase was recovered by centrifugation (12,000 *g* for 20 min), and for every 1 mL, 170 μL of 10% CTAB was added. After 30 min of incubation at 65 °C, an equal volume of phenol, chloroform, and isoamyl alcohol (25:24:1) was added again. The aqueous phase was recovered by centrifugation (12,000 *g* for 20 min) and the nucleic acids were precipitated with 0.8 volumes of isopropanol at −20 °C for 1 h. The mDNA was recovered by centrifugation at 12,000 *g* for 20 min, re-suspended in 200 μL of TE buffer (20 mM Tris–HCl, 1 mM EDTA, pH 8) and analysed by 1% agarose gel electrophoresis. mDNAs were kept at −80 °C until further analysis.

### 2.3. Shotgun Metagenomics Analysis

NGS was performed by the BASECLEAR company (Leiden, The Netherlands). Libraries were sequenced as 300 bp paired-end reads on an Illumina MiSeq. Adapter sequences and low-quality bases were trimmed using trimmomatic [17] with a minimum average quality score threshold of 20 in a 4 bp sliding window. Raw and trimmed read quality was assessed using FastQC. A taxonomic profile was generated for each sample using Kraken2 v2.1.3 [18] with the PlusPFP RefSeq database available at https://benlangmead.github.io/aws-indexes/k2 (accessed on 22 January 2024). Genus- and species-level relative abundances were estimated from Kraken2 taxonomic profiles using Bracken v2.9 [19]. Functional profiling was performed using HUMAnN3 [20] with built-in UniRef50 and UniRef90 databases. Identified gene families were summarised as MetaCyc Reactions [21], KEGG Orthogroups (KOs) [22], PFAM domains [23], EggNOG clusters of orthologous groups (COGs) [24] and Gene Ontology (GO) [25,26] gene sets using the humann_regroup_table function and built-in mappings available with HUMAnN3 v3.9.0. Abundance estimates for gene families were normalised as copies per million mapped reads (CoPM) for comparison between samples.

Metagenomic assembly of trimmed reads was performed using metaSPAdes v3.15.4 [27], and assembly quality was evaluated using MetaQUAST v5.0.2 [28]. Resulting contigs larger than 1000 bp were binned to retrieve MAGs using MetaWRAP v1.3.2 [29] with MaxBin 2.0 [30], MetaBAT 2 [31] and CONCOCT v1.1.0 [32]. MAGs were checked for completeness and contamination using CheckM2 v1.0.1 [33]. The ‘bin_refinement’ module from MetaWRAP was used with parameters -c 50 -x 10 to retain only bins with a minimum completeness of 50% and a maximum contamination of 10% (i.e., medium-quality and better MAGs) [34]. MAGs were compared using dRep v3.5.0 to determine if duplicate genomes had been assembled across samples, but no bins exceeded the primary clustering threshold of 90% MASH average nucleotide identity. MAGs were taxonomically classified using ‘classify_wf’ and sets of conserved marker genes (53 archaeal and 120 bacterial) in GTDB-Tk v2.4.0 [35], with release 220 of the GTDB database [36] (accessed on 24 April 2024). A phylogenetic tree for the 93 bacterial MAGs was generated based on the alignment of the concatenated 120 bacterial marker genes using ‘infer’ from GTDB-Tk and plotted using the R package ggtree v3.11.2 [37]. MAGs were functionally annotated using Prokka v1.14.5 [38] with the ‘annotate_bins’ module from MetaWRAP.

### 2.4. Cloning, Expression and Substrate Scoping of Metagenomic Aminotransferases Class III

The putative aminotransferase class III genes (ATs) from Maras3 were amplified by PCR from the respective metagenome. The amplicons were confirmed in agarose gel electrophoresis and recovered from the gel. ATs cloning was performed in pJL1c vector and transformed into chemically competent *E. coli* DH5-alpha (for plasmid maintenance) and Rosetta (for protein expression); they were stored in glycerol stocks 25% at −80 °C. The AT sequences were confirmed by sequencing (Eurofins Genomics), and positive TAs were labelled with a respective pQR number (internal plasmid construct identifier, as shown in Supplementary Table S2).

To identify cloned AT gene sequences in the Maras3 metagenomic assembly, gene sequences were searched against the entire Maras3 assembly using blastn from the BLAST+ package (v2.16.0) [39]. Hits were filtered for identity >95% and length >500. Contigs containing AT gene BLAST hits which had been assembled into MAGs were classified using the taxonomy previously assigned to the MAG using GTDB-Tk, and unbinned contigs were classified with CAT_pack contigs (v6.0) using the same GTDB database (release 220) as for MAG classification. Contig relative abundances were estimated by aligning all reads onto the Maras3 assembly using bowtie2 (v2.5.4) [40] followed by Coverm contig (v0.7.0) [41].

ATs were expressed in 10 mL of TB broth with kanamycin 50 μg/mL at 37 °C and 250 rpm and grown until an OD_600nm_ ~1 was reached, then induced with isopropyl β-D-1-thiogalactopyranoside (IPTG) 0.1 mM final concentration and further incubated at 25 °C for 15 h. The cells were harvested by centrifugation at 12,300 *g* at 4 °C for 30 min. The cell pellet was re-suspended with 500 μL of lysis buffer (HEPES 50 mM, pH 7 supplemented with 0.2 mM PLP) and sonicated for 10 cycles (10 s ‘ON’ and 10 s ‘OFF’) at 15 μA amplitude. The lysates were then centrifuged 20,800 *g* at 4 °C for 30 min. The soluble crude lysate (supernatant) was collected and used for further activity assay.

AT substrate scoping was assayed for the following aromatic and aliphatic substrates: Acetophenone, Benzaldehyde, Pyruvate, D-xylose, Furfural, Methyl furfural, Hydroxymethyl furfural, 2-phenyl-2-butanone, Acetoin, 2-ketobutyric acid and α-ketoglutaric acid. The reactions were carried out by mixing 20 μL of AT crude lysate with 180 μL of substrate mix containing 10 mM substrate, 25 mM 2-(4-nitrophenyl)ethan-1-amine as amino donor, and 0.2 mM PLP in buffer HEPES 50 mM pH 7. The reactions were carried out in sealed 96-well plates and incubated at 37 °C for 24 h. The positive reactions led to the formation of an orange-red coloration [42]. A negative control reaction was also performed following the above procedure without the addition of enzyme.

## 3. Results

### 3.1. Sampling Site and mDNA Extraction

Maras salterns are formed by a group of man-made salt ponds built in pre-Inca times and are currently exploited for salt commercialisation. They are located 3030 m above sea level in Urubamba town (Cusco, Peru) where the dry season is from May to November and the rainy season from December to April. Over the year, the temperature ranges from −8 °C at night to 20 °C during the day. These salterns are characterised by their alkalinity, low oxygen concentration, and salinity above 25% in both the emergent water and crystallised ponds [15,16]. Pilluana salterns are natural and unexploited salt deposits, with a salinity of around 20%. They are located in the Amazon rainforest at 200 m above sea level in the basin of the Huallaga River in Picota town (San Martin, Peru) and are fed by the thermal waters of Picuro Wuasi. In Picota, the dry season is from June to September and the rainy season is from October to May; it has a tropical climate with temperatures from 17 to 40 °C. For mDNA extraction, two soil samples were taken from Maras (Maras3 and Maras6), and one from Pilluana (Pilluana). At least 500 ng of mDNA was isolated per sample with a concentration >25 ng/μL. The 1% agarose gel electrophoresis analysis showed that the extracted mDNAs were of good quality and adequate for sequencing (Supplementary Figure S1).

### 3.2. Diversity and Taxonomic Composition

The reads generated from each sample were trimmed and classified using Kraken2 v2.1.3 with the PlusPFP RefSeq database containing reference sequences of archaea, bacteria, viruses, humans, protozoa, fungi, and common plasmids and vectors. The number of trimmed reads from Maras3, Maras6 and Pilluana samples was 8,099,362 (42.18% classified), 8,235,307 (45.33% classified), and 6,998,669 (47.24% classified), respectively. Alpha diversity (within samples) was calculated with Shannon, Simpson and Berger–Parker indices. The metrics indicated slightly higher diversity in Maras samples compared to Pilluana (Supplementary Table S3).

**Figure 2:**
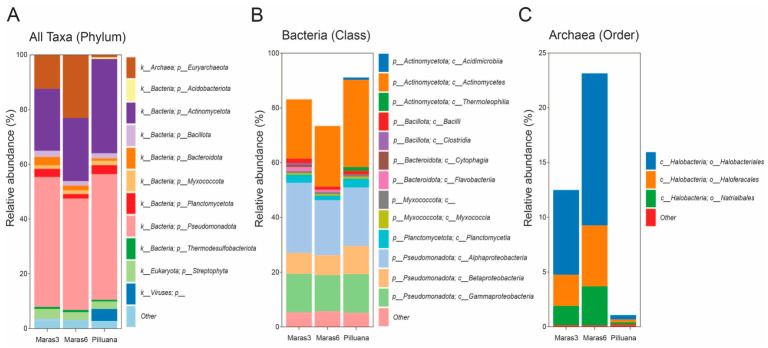
Taxonomic classification and relative abundances for classified reads in shotgun metagenomic sequencing of Peruvian saltern soil samples generated by Kraken2 v2.1.3. (**A**) Phylum level, (**B**) class level for bacterial phyla and (**C**) order level for archaea (phylum *Euryarchaeota*). Phyla and classes comprising <0.5% and orders <0.1% are shown as ‘Other’.

Phylum relative abundance estimation (Figure 2A) revealed that classified reads in all samples were mostly derived from bacterial phyla, with *Pseudomonadota* (~45%) and *Actinomycetota* (~30%) being the most dominant. However, Maras samples also included a notably large proportion of reads assigned to the archaeal phylum *Euryarchaeota* (Maras3, 12.42% and Maras6, 23.09%) compared to the Pilluana sample (0.91%). In the Pilluana sample, 4.37% of reads were classified as belonging to an unknown virus phylum. All of them belonged to *Alphabaculovirus* species with the highest percentage classified as *Alphabaculovirus aucalifornicae* (4.26%). Bacterial reads at class level (Figure 2B) showed *Actinomycetes* (~25%), followed by *Alphaproteobacteria* (~20%), *Gammaproteobacteria* (~15%), and *Betaproteobacteria* (~10%) as the most predominant in all samples. *Clostridia* and *Cytophagia* classes were detected in Maras3 at >0.5% abundance, but not in Maras6 and Pilluana. The *Flavobacteriia* class was detected in Maras samples at >0.5% abundance, but not in the Pilluana sample. *Acidimicrobiia* and *Thermoleophilia* classes were detected in the Pilluana sample at >0.5%, but not in the Maras samples. These classes comprising <0.5% relative abundance were classified as ‘Other’. As described above, both Maras samples presented a remarkably higher abundance of archaea than the Pilluana sample. The majority of archaeal reads detected belonged to the class *Halobacteria* and the order *Halobacteriales* (Figure 2C).

**Figure 3:**
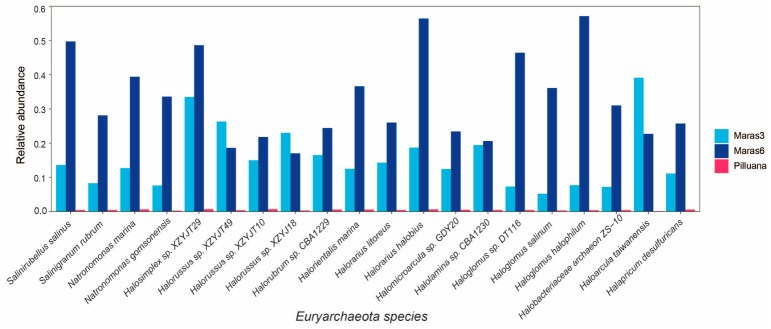
Relative abundances for the top 20 *Euryarchaeota* species ranked by mean abundance across the three Peruvian saltern soil samples. Data were analysed using Kraken2 v2.1.3/Bracken v2.9.

Archaeal species with the highest abundance identified in Maras samples were *Haloglomus halophilum*, *Halorarius halobius*, *Salinirubellus salinus*, *Halosimplex* sp. XZYJT29, *Haloglomus* sp. DT116, *Natronomonas marina*, *Haloarcula taiwanensis*, *Halorientalis marina*, *Haloglomus salinum*, *Natronomonas gomsonensis*, *Halobacteriaceae archaeon* ZS−10 and *Salinigranum rubrum* (Figure 3). These species were also identified in the Pilluana sample but at negligible relative abundance.

**Figure 4:**
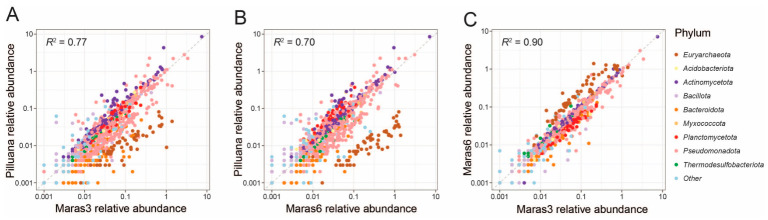
Pairwise comparisons of genus-level relative abundances for bacterial and archaea (*Euryarchaeota*) phyla between Peruvian saltern soil samples. (**A**) Maras3 vs. Pilluana, (**B**) Maras6 vs. Pilluana, and (**C**) Maras3 vs. Maras6. An arbitrary pseudocount of 0.001 was added to all values to facilitate plotting on a log axis. The dots are coloured by phylum as shown in Figure 2A. Data were analysed using Kraken2 v2.1.3/Bracken v2.9.

Comparison of genus-level relative abundances between the three samples showed several differentially abundant genera, particularly a number from the phylum *Euryarchaeota* (Figure 4). Comparable profiles were observed between either of the Maras samples and the Pilluana sample (R^2^ = 0.7) (Figure 4A,B). As expected, both Maras samples exhibited a highly similar profile of genus-level abundances as shown by linear regression analysis (R^2^ = 0.9) (Figure 4C).

### 3.3. Functional Profiling

**Figure 5:**
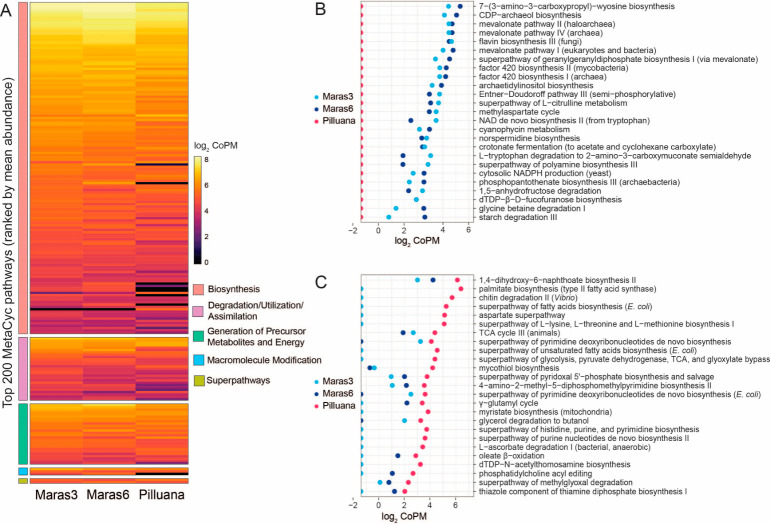
Functional profiling in shotgun metagenomic sequencing of Peruvian saltern soil samples using HUMAnN 3.9.0. (**A**) Heatmap of log2 normalised abundance expressed as copies per million mapped reads (CoPM) for the top 200 MetaCyc pathways. (**B**) Maras and (**C**) Pilluana top 25 MetaCyc pathways with the highest abundance. Pathways were grouped by top-level MetaCyc category and ordered by mean abundance within groups.

Trimmed metagenomic reads were mapped to UniRef90 clusters using HUMAnN 3.9.0 to quantify gene families present in each sample. There were clear differences between Maras and Pilluana samples for the most abundant pathways when gene families were summarised at the MetaCyc pathway level (Figure 5). Biosynthetic pathways were the most predominant across all the samples. Higher abundances for the top 200 MetaCyc pathways were observed in Maras samples, mainly in Maras6 (Figure 5A). To identify differentially abundant pathways between Maras and Pilluana samples, all pathways with a mean abundance (expressed as CoPM) <1 were filtered out. Then, the log2 ratio of the mean abundance at Maras to the abundance at Pilluana was calculated. Similar profiles were observed for both Maras samples related to the top 25 differentially abundant pathways (Figure 5B). However, these pathways were not found in the Pilluana sample. A number of pathways specific to archaea were abundant in Maras samples, as expected from the taxonomic profiling analysis presented in Figure 2A. Abundant pathways in Maras samples also included those related to the degradation of compatible osmolytes such as glycine betaine. Similarly, the top 25 differentially abundant pathways in the Pilluana sample were considerably different from those in the Maras samples, in which some were at a lower abundance and others were not found (Figure 5C). The top Pilluana pathways were mainly associated with fatty acid biosynthesis and oxidation.

Compatible osmolytes are key metabolites in microorganisms from saline environments. Analysis of pathways and Pfam domains of compatible osmolytes such as ectoine and glycine betaine revealed their higher abundance in both Maras samples than in the Pilluana sample (Supplementary Figure S2). Likewise, the ectoine biosynthesis pathway was more abundant than the degradation one and glycine betaine transporters were found to be abundant.

### 3.4. Metagenome-Assembled Genomes (MAGs)

Metagenomic reads from each sample were assembled separately into contigs using metaSPAdes. Contigs <1000 bp were filtered out for binning of the contigs to produce MAGs. The remaining contigs were binned using metaWRAP to produce a refined set of bins from multiple independent binning algorithms (MaxBin2, MetaBAT2 and CONCOCT). Bins were refined using the ‘bin_refinement’ module in metaWRAP, with minimum completeness of 50% and maximum contamination of 10%. These percentages were determined by CheckM v1.0.1, which uses conserved marker genes to assess bin quality.

As a result of this analysis, 96 MAGs (completeness > 50% and contamination < 10%) were taxonomically classified based on concatenated alignments of marker gene sets (53 archaeal and 120 bacterial) using GTDB-Tk. The number of retrieved MAGs from Maras3, Maras6 and Pilluana samples was 25, 24 and 47, respectively. Three MAGs, one from each sample, were archaeal and assigned to the genera *Halorussus*, *Halorubellus* and *Nitrosotenuis* (Supplementary Table S4). The most complete archaeal MAG was that of *Halorubellus* with 82.61% completeness, 1.55% contamination, size of 2.60 Mb and 66% GC content.

**Figure 6:**
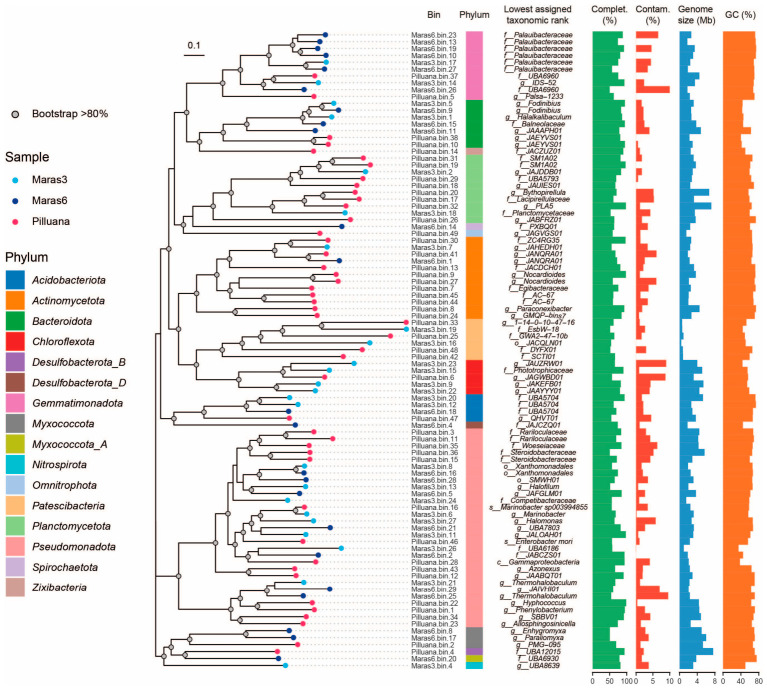
Phylogenetic tree of 93 bacterial MAGs compiled from assembled metagenomic reads following binning of metaSPAdes-assembled contigs using metaWRAP. MAGs with >50% completion and <10% contamination determined by CheckM v1.0.1 are shown. The unrooted tree was produced using the ‘classify_wf’ workflow in GTDB-Tk based on a concatenated alignment of 120 bacterial marker genes. Tips are coloured by the sample from which each MAG was binned, and nodes with >80% bootstrap support are marked. The taxonomic assignments from GTDB-Tk ‘classify_wf’ and bin stats calculated using CheckM v1.0.1 are also shown.

A phylogenetic tree, showing taxonomic assignments from GTDB-Tk and bin stats from CheckM, was constructed for the remaining 93 MAGs, which belonged to bacteria (Figure 6). Most MAGs were classified within the phyla *Psudomonadota*, *Actinomycetota*, *Planctomycetota*, and *Gemmatimonadota*. MAGs belonging to the order *Xanthomonadales*, family *Palauibacteraceae*, genera *Fondinibius* and *Nocardioides*, and species *Marinobacter* sp003994855 and *Enterobacter mori* were also retrieved. The MAG with the highest completeness (97.77%) was from the Pilluana sample (Pilluana.bin.9); this was classified within the genera *Nocardioides* (1.11% contamination, 3.69 Mb and 72.3% GC content). The largest retrieved MAG (7.61 Mb) was also from the Pilluana sample (Pilluana.bin.4), which was classified within the UBA12015 family (93.52% completeness, 2.26% contamination and 70.8% GC content).

MAGs were functionally annotated using Prokka v1.14.5 and the ‘annotate_bins’ module from metaWRAP v1.3.2. MAGs containing annotated genes similar to the selected genes involved in the metabolism and transport of the compatible solutes ectoine and glycine betaine were analysed (Supplementary Figure S3). Genes involved in ectoine synthesis and degradation along with genes implicated in glycine betaine synthesis and transport were mainly encoded by MAGs from Maras samples. Most of the MAGs encoding these genes were classified within the phylum *Pseudomonadota*.

### 3.5. Metagenomic Aminotransferases

Microbial aminotransferases (ATs) are a diverse group of pyridoxal-5′-phosphate (PLP)-dependent enzymes that play a fundamental role in nitrogen metabolism by catalysing the reversible transfer of an amino group from a donor molecule to an acceptor. This process is essential for the biosynthesis of amino acids and various secondary metabolites. Based on their phylogeny and substrate acceptance, ATs are classified in classes I-II (PF00155), III (PF00202), IV (PF00266), V (PF01063) and DegT/DnrJ/EryC1/StrS (PF01041) [43]. In the present study, the analysis of AT abundance across three sampling sites (Maras6, Maras3, and Pilluana) revealed distinct distribution patterns among five identified Pfam families (Supplementary Figure S4). The PF00155 family was the most prevalent across all sites, showing a downward trend in abundance from Maras6 (~360 CoPM) to Pilluana (~310 CoPM). In contrast, PF01041 represented the least abundant family, uniquely exhibiting an inverse relationship where Pilluana recorded the highest values. Whilst Maras6 generally displayed the highest CoPM values for most families (including PF00202, PF00266, and PF01063), Pilluana consistently showed the lowest relative abundance for these same categories. Notably, Maras3 and Maras6 exhibited nearly identical levels for the PF00202 family, suggesting localised similarities in AT profiles between these two sites.

**Figure 7:**
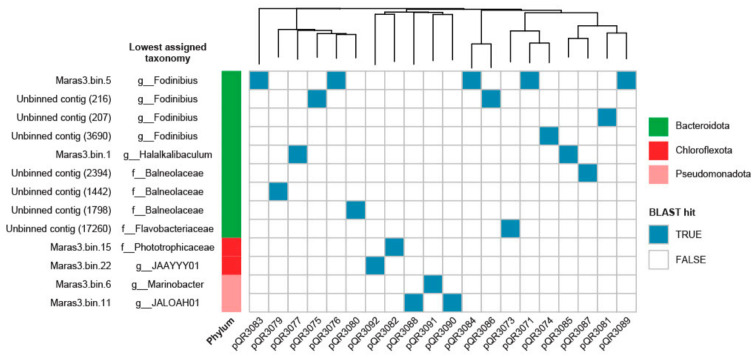
Identification and phylogenetic analysis of cloned ATs class III in the Maras3 metagenomic assembly; gene sequences were searched against the entire Maras3 assembly using contigs containing AT genes. BLAST hits that had been assembled into MAGs were classified using the taxonomy previously assigned to the MAG using GTDB-Tk v2.4.0, and unbinned contigs were classified with CAT_pack contigs.

A total of 20 putative metagenomic ATs class III were successfully cloned from the Maras3 site (Figure 7). The AT phylogenetic analysis identified diverse microbial taxa across three major phyla: *Bacteroidota*, *Chloroflexota*, and *Pseudomonadota*. Remarkably, twelve ATs were identified within the Maras3 MAGs, whilst the remaining were in unbinned contigs. BLAST v2.16.0 hit screening against a panel of ATs revealed highly distinct, non-overlapping distribution profiles across the taxa. Hierarchical clustering grouped the ATs into distinct clades based on their presence or absence profiles. Taxa belonging to *Bacteroidota* constituted the largest portion of the recovered binned and unbinned contigs, where 45% of ATs were represented by the genus *Fodinibius*, which exhibited the highest diversity and tested positive for five distinct ATs across disparate clusters of Maras3.bin.5 (pQR3083, 3076, 3084, 3071, and 3089). All members of the family *Balneolaceae* were found in unbinned contigs. The *Chloroflexota* phylum was represented by Maras3.bin.15 (family *Phototrophicaceae*), which was selectively associated with pQR3082, whilst Maras3.bin.22 was uniquely clustered with pQR3092 (genus *JAAYYY01*). The ATs from *Pseudomonadota* were represented by Maras3.bin.6 (genus *Marinobacter*) and Maras3.bin.11 (genus *JALOAH01*) bins, which showed higher homology levels.

**Figure 8:**
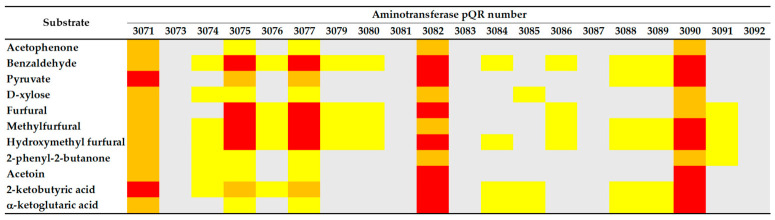
Heatmap of the substrate scoping of ATs class III from Maras3 towards aldehydes and ketone substrates: red (strong activity), orange (medium activity), yellow (weak activity) and grey (no activity detected).

The substrate scoping profile of the screened metagenomic ATs class III revealed a high degree of functional diversity, with activity levels ranging from strong to negative across eleven distinct aldehyde and ketone substrates (Figure 8 and Figure S5). Remarkably, ATs pQR3082 and 3090, which belong to the genus *Fodinibius* and *JALOAH01*, respectively, emerged as the most versatile and potent biocatalysts, exhibiting strong activity (red) against nearly all tested substrates. In contrast, five ATs showed no detectable activity across the entire panel, suggesting a high degree of substrate selectivity or a poor compatible AT expression under the tested conditions. Furfural and its derivatives were generally well accepted by most TAs, predominantly yielding medium to strong reactions. Notably, the ATs pQR3075 (genus *Fodinibius*) and pQR3077 (genus *Halalkalibaculum*) showed more specialised activity towards aromatic aldehydes.

## 4. Discussion

### 4.1. Peruvian Saline Environments: Maras and Pilluana Salterns

Saline environments in South America have outstanding environmental conditions influenced by their geographic location, making them unique niches for extremophiles [11]. These environments can be classified as thalassohaline or athalassohaline. Thalassohaline environments have a marine origin, where the most abundant salt is sodium chloride and the pH values are from neutral to alkaline. Athalassohaline environments do not have a marine origin and are predominantly in tropical areas with high temperatures and solar radiation, and different salts may be present [44]. Both Maras and Pilluana salterns can be classified as thalassohaline environments but with different geological origins and characteristics.

Maras salterns are in the Andean region, which has a temperate climate. They consist of around 3000 unconnected shallow ponds forming terraces along the side of the mountain. These ponds have been made by humans and are currently exploited for commercial salt production. Each pond is fed by hypersaline water coming from a subterranean spring, which originated from an ocean that covered this region millions of years ago and remained on land during the formation of the Andes mountains [15,16]. In contrast, Pilluana salterns are located in the Amazon region, which has a tropical climate. They were formed millions of years ago when this area was covered by an epicontinental sea that evaporated over the years, leaving salt deposits that were buried under layers of sediment. Tectonic movements and volcanic activity in this region contributed to the formation of emergent saline structures, which, in combination with minerals such as iron, calcium and others, led to the formation of pink, white and black salt [45]. Unlike the Maras salterns, the Pilluana salterns remain in their natural form without human exploitation for commercialisation.

### 4.2. Soil Samples for Metagenomic Studies

Soils are considered among the richest ecosystems because of their great microbial diversity, which remains relatively unexplored [46,47]. Soil samples can be challenging for a metagenomic analysis due to their heterogeneous properties and composition [47]. They are characterised by the presence of ions, heavy metals, and humic substances (organic contaminants), which interfere with mDNA extraction, sequencing and manipulation [48,49]. In this study, sequential pre-lysis washes of the soil samples were carried out, which promotes soil dispersion and removal of salts, metals and organic contaminants [48,50]. After testing different pre-lysis washing conditions, a ratio of soil:washing buffer pH 8 of 1:1.5 and repeated 4 times enabled the recovery of mDNA at high concentrations and purity.

### 4.3. Microbial Diversity and Taxonomic Composition of Maras and Pilluana Salterns

Alpha diversity indices indicated more diversity in both Maras samples than in the Pilluana sample, which could be explained by the different environmental features of each saltern such as geological characteristics, location, altitude, temperature, salt and mineral concentrations. *Pseudomonadota* was the main bacterial phylum determined across our samples. Similar results from different hypersaline environments around the world were also reported, such as Cabo Rojo, Puerto Rico [4]; Karak Salt Mine, Pakistan [10]; Solar de Ascotán, Chile [11]; Yilgarn Craton brine lakes, Australia [14]; Namib Desert salt pans, Namib [51]; and Radha- Krishna saltern, India [52]. Within this phylum, *Alphaproteobacteria*, *Gammaproteobacteria* and *Betaproteobacteria* classes were the most abundant at close percentages in all our samples; these results were also in agreement with other similar studies [10,14,51]. These results confirm that these types of bacteria have developed unique mechanisms of adaptation to different extreme environments; this adaptation capability is regardless of the geographical location of the saline environment, as demonstrated in our study where Andean and Amazonian microbial distributions are compared.

*Actinomycetota* was the second most abundant bacterial phylum in our samples; however, it has been reported at low percentages in other saline environments worldwide [4,14,52]. The predominant class found within this phylum, and with the highest relative abundance within our samples, was *Actinomycetes*. These bacteria are commonly found in soil samples as they play a critical role in soil processes (e.g., decomposition and ammonium fixation). Several halotolerant and halophilic *Actinomycetes* were uncovered through metagenomic studies; these bacteria are highly relevant as producers of a variety of secondary metabolites including antimicrobials [53]. In addition, the *Acidimicrobiia* and *Thermoleophilia* classes were found in the Pilluana sample. These bacterium classes have been scarcely cultivated, poorly characterised and mainly studied by metagenomic means [54,55]. These bacteria have adapted to high salt concentrations and temperatures, acidic conditions and high iron salt concentrations, features of the Pilluana sample.

The bacterial phylum *Bacteroidota* was detected at low abundance in our samples in comparison with previous studies in saline environments [4,10,11]. Within this phylum, *Flavobacteriia* and *Cytophagia* classes were identified in the Maras samples (>0.5%). They are commonly described as polysaccharide-degrading enzymes and protease producers [56].

The Archaea domain is highly diverse and ubiquitous in nature; these microorganisms play an important role in ecology and are relevant in the biotechnology sector. In this study, the archaeal phylum *Euryarchaeota* was remarkably more abundant in Maras samples than in the Pilluana one, where it was almost negligible, with *Halobacteriales* being the predominant order. Studies have reported that high-salinity environments (above 30%) present higher archaea abundance [4,10,52]. Thus, these findings might be associated with the higher salt concentration in Maras salterns than in Pilluana, which is also supported by their higher altitude [11]. Among the most abundant archaeal species found in Maras samples, as also reported in other studies, were *Haloglomus halophilum*, *Halorarius halobius*, *Natronomonas marina* and *Haloglomus salinum* (isolated in 2023, China) [57]; *Halosimplex* sp. XZYJT29 and *Haloglomus* sp. DT116 (isolated in 2024, China) [58,59]; and *Halobacteriaceae archaeon* ZS−10, reclassified as *Salinigranum marinum* in 2023 [60].

### 4.4. Functional Profiling of Maras and Pilluana Salterns: Metabolic Pathways and Metabolites

Metagenome mining is a key approach for biocatalyst discovery that enables unique and unexplored single enzymes and metabolic pathways to be revealed. For example, a recent study demonstrated the huge potential of our Maras and Pilluana metagenome samples to expand the knowledge regarding the understudied ‘split’ transketolases, which are catalytically analogues to full-length transketolases and widely used in industry [61].

Herein, MetaCyc metabolic pathways from both Maras samples were very similar, but considerable differences were observed between Maras and Pilluana samples. These findings might, to some extent, reflect the higher abundance of archaea in Maras samples since several of the top pathways identified belong to archaea. From the Maras samples, the most prevalent metabolic pathways, particularly archaeal Mevalonate pathways II and IV, are involved in the biosynthesis of isoprenoids that act as cofactors, UV protectors, antioxidants and regulators of membrane fluidity [62,63]. Abundant non-exclusive archaeal pathways identified in Maras included factor 420 biosynthesis I, norspermidine biosynthesis and starch degradation III.

Regarding the metabolic pathways predominately found in the Pilluana sample, the 1,4−dihydroxy−6−naphthoate biosynthesis II pathway, also found in the Maras samples in high abundance, is involved in the synthesis of quinones (reversible redox component of the electron transfer chain). Most pathways described in the Pilluana sample mainly belong to bacteria and are involved in fatty acid biosynthesis (e.g., palmitate biosynthesis and superpathway of fatty acid biosynthesis) and oxidation (e.g., oleate β−oxidation). Many of these enzymes and intermediates are useful for producing fatty acid-derived chemicals [64]. In addition, biosynthetic pathways related to amino acids and vitamin B, and degradation pathways such as chitin degradation II (chitinases) and glycerol conversion to butanol, were abundant in Pilluana. The efficient microbial production of amino acids and [65] vitamin B is of value in food industry [66], whilst chitinases have applications in food industry, medicine and agriculture [67].

Functional information about compatible osmolytes such as glycine betaine and ectoine was also analysed since they are essential in halophilic archaea and bacteria for adaptation to extreme saline environments. Compatible osmolytes can be biosynthesised or absorbed from the environment, overcoming osmotic, temperature and UV stress in halophiles [68]. The higher abundance of glycine betaine and ectoine pathways in Maras than in Pilluana samples likely reflects their higher stressful environmental conditions, especially the higher salt concentrations, as the levels of these osmolytes are highly regulated by external salt levels, which trigger their biosynthesis and uptake. Surprisingly, the Maras and Pilluana samples had very low and negligible abundances, respectively, of the ectoine degradation pathway. We hypothesise that this pathway exists predominantly in non-halophilic bacteria that are unable to synthesise ectoine. The abundance of glycine betaine transporters suggests that in addition to being synthesised, it is absorbed from the environment [69].

### 4.5. Archael and Bacterial MAGs and Their Biotechnological Potential

The two archaeal MAGs retrieved from Maras were identified as *Halorussus* and *Halorubellus*, which include genes encoding biotechnologically relevant enzymes such as glycosidases, proteases, carboxypeptidases, esterases and nitrite reductases [70,71]. The unique archaeal MAG retrieved from Pilluana was identified as *Nitrosotenuis*, described as an ammonia-oxidizing thermophilic archaea with a key role already established in wastewater treatment [72].

Bacterial MAGs retrieved from our samples were classified within the families *Palauibacteraceae* and *Competibacteraceae*. MAGs belonging to the bacteria genera *Fodinibius*, *Nocardioides*, *Marinobacter*, *Halomonas*, *Azonexus*, *Hyphococcus*, *Enhygromyxa* and *Paraliomyxa* were also retrieved. Several of these bacteria have been identified only by metagenomics in marine and other salt-related environments and associated with the production of industrially relevant chemicals [73,74,75,76].

One MAG at species level was retrieved and classified as *Enterobacter mori*, which is a plant growth-promoting bacteria able to survive drought stress, thereby improving agricultural yields [77]. The rest of our MAGs, including the largest MAG classified within the UBA12015 family, have been reported in different metagenomic studies worldwide. They have been assigned to a taxonomic classification without genomic representation and further studies will be required to better understand them [78].

### 4.6. Biotechnological Potential of Metagenomic Aminotransferases

The distinct distribution and functional versatility of ATs observed in this study highlight how environmental niches shape microbial metabolic potential, offering a diverse genetic reservoir for biotechnological innovation [79]. The dominance of the PF00155 family (classes I-II) across all sampling sites suggests a core evolutionary conservation of these enzymes, likely due to their essential role in primary nitrogen metabolism and the biosynthesis of basic amino acids. However, the site-specific fluctuations in family abundance, particularly between the Maras and the Pilluana sites, might reveal a deeper level of ecological adaptation. The higher abundance of most AT families in the Maras sites may be a direct response to the physiological demands of halophilic microbial communities. In such high-salinity environments, robust nitrogen metabolism is critical for the synthesis of nitrogen-based compatible solutes, which are essential for maintaining osmotic balance and cellular integrity.

Of particular interest for biotechnology are the high prevalence and stability of the PF00202 family (ATs class III, also called ω-transaminases) at the Maras3 site. Extremophilic ATs class III are among the most sought-after biocatalysts in the pharmaceutical and fine chemical industries due to their unique ability to accept ω-amino acids and bulky amines as substrates [80]. These enzymes are critical for the asymmetric synthesis of chiral amines, which serve as building blocks for approximately 50% of modern pharmaceutical drugs [81]. The near-identical CoPM values for ATs class III at Maras3 and Maras6 suggest a localised metabolic specialization that favours these enzymes. Most of these novel metagenomic ATs class III were from the family *Balneolaceae*, including the genus *Fodinibius*. To date only eight *Fodinibius* species have been identified and characterised, all of them being isolated from hypersaline environments [82].

From a bioprospecting perspective, enzymes sourced from such hypersaline, high-altitude Andean environments are often extremozymes possessing inherent thermostability and tolerance to organic solvents, traits that are indispensable for industrial-scale biocatalysis where reaction conditions are often harsh [83]. The broad substrate promiscuity of pQR3082 and pQR3090, especially toward industrially relevant aldehydes like furfural, identifies them as robust candidates for green chemistry applications. Furfural, a key biomass-derived platform chemical, was generally well accepted across the ATs class III, suggesting that these metagenomic ATs are well suited for biorefinery processes aimed at converting renewable feedstocks into value-added chemicals.

## 5. Conclusions

This comparative metagenomic study reveals the taxonomic and functional profiles of microorganisms from the Peruvian salterns of Maras and Pilluana. Although these unique salterns have a similar ancient marine origin, our metagenomic analysis demonstrates that their different geographical locations and specific environmental conditions have led to different microbial populations. Maras salterns are more microbially diverse with a higher abundance of archaea and halophilic bacteria, which is consistent with their more variable extreme environmental conditions. Functional analysis from both salterns reveals different metabolic pathway abundances, providing insights into microbial adaptation to their respective habitats. The integration of metagenomic distribution data with functional activity profiles provides a powerful framework for enzyme discovery. Here, highly promiscuous ATs class III were identified, offering a novel and unique alternative to the current non-extremophilic counterparts. By identifying the biotechnological drivers behind the abundant enzyme families, we can better target specific ecological niches for the recovery of enzymes with desired industrial traits. These findings not only advance our understanding of microbial distribution in extreme environments but also expand the biocatalytic toolbox, offering better enzymatic alternatives than the current ones. Further research is merited to explore the enormous taxonomic and functional richness of these exceptional environments, as well as ATs’ full biochemical characterization and their biotechnological application.

## Data Availability

MAG assemblies were deposited in NCBI under the BioProject ID PRJNA1184742.

## Supplementary Materials

The following supporting information can be downloaded at: https://www.mdpi.com/article/10.3390/microorganisms14071595/s1, Figure S1. Agarose gel electrophoresis (1%) showing the extracted mDNA of soil samples from Peruvian salterns. (A) Maras3, (B) Maras6 and (C) Pilluana3. Lanes: 1, mDNA; and MW, molecular weight marker. Figure S2. Functional analysis of ectoine and glycine betaine in shotgun metagenomic sequencing of Peruvian salterns soil samples using HUMAnN 3.0. Normalised abundance of (a) MetaCyc pathways involved in ectoine biosynthesis and degradation; and (b) Pfam domains involved in ectoine biosynthesis and glycine betaine transport. Figure S3. Heatmap showing the presence/absence of selected genes involved in ectoine synthesis and degradation, as well as glycine betaine synthesis and transport pathways in MAGs from Peruvian salterns samples. MAGs were functionally annotated using the ‘annotate_bins’ module in metaWRAP. MAGs (bins) are labelled with phylum (assigned by GTDB-Tk) and sample, as in Figure 7. Figure S4. Distribution of metagenomic ATs in all site samples. Figure S5. Aminotransferase substrate scoping assayed for the following aromatic and aliphatic substrates: (1) Acetophenone, (2) Benzaldehyde, (3) Pyruvate, (4) D-Xylose, (5) Furfural, (6) Methyl furfural, (7) Hydroxymethyl furfural, (8) 2-phenyl-2-butanone, (9) Acetoin, (10) 2-ketobutyric acid and (11) a-ketoglutaric acid. The reactions were carried out by mixing 20 mL of AT crude lysate with 180 mL of substrate mix containing 10 mM substrate, 25 mM 2-(4-nitrophenyl)ethan-1-amine as amino donor, and 0.2 mM PLP in buffer HEPES 50 mM pH 7. The reactions were carried out in sealed 96-well plates and incubated at 37 °C for 24 h. ATs from A1 to B8, pQR number as in Table S3. Negative control reactions in B9 (no enzyme) and B10 (no amino donor) Table S1: Parameters of sample sites from Peruvian salterns. Table S2: Gene sequences of the putative metagenomic aminotransferases class III isolated from Maras3. Plate position corresponds to the bioprospecting prospecting experiments (as shown in Figure 6 and Figure S4). pQR numbers correspond to the internal identifier codes. Table S3: Alpha diversity metrics for the Peruvian salterns samples. Table S4: Archaeal MAGs recovered from Peruvian salterns samples.
