# Supplementary material for: Taxonomic and Functional Comparative Metagenomics of Peruvian Salterns: Insights into Microbial Communities and Aminotransferase Potential
Source: Microorganisms. 2026 Jul 22;14(7):1595. doi: 10.3390/microorganisms14071595 (PMC13413810; doi:10.3390/microorganisms14071595)
Supplement: Supplementary file 1 [file microorganisms-14-01595-s001.zip › microorganisms-4426320-supplementary.pdf]

Supplementary Information

Article

Taxonomic and Functional Comparative Metagenomics of Peruvian Salterns: Insights into Microbial Communities and Aminotransferase Potential

Carol N. Flores-Fernández[1,2], Thomas K. Hiron[1], Dragana Dobrijevic[3], Amparo I. Zavaleta[2], Jack W.E. Jeffries[4], Chris A. O'Callaghan[1], Gary J. Lye[3], John M. Ward[3,*], Max Cárdenas-Fernández[5,6,*]

1 Centre for Human Genetics, Nuffield Department of Medicine, University of Oxford, Roosevelt Drive, Oxford OX3 7BN, UK; nathali.flores@well.ox.ac.uk (C.N.F.-F.); thomas.hiron@dpag.ox.ac.uk (T.K.H.); chris.ocallaghan@ndm.ox.ac.uk (C.A.O.)
2 Laboratorio de Biología Molecular, Facultad de Farmacia y Bioquímica, Universidad Nacional Mayor de San Marcos, Lima 15001, Peru; azavaletap@unmsm.edu.pe
3 Department of Biochemical Engineering, The Advanced Centre for Biochemical Engineering, University College London, London WC1E 6BT, UK; draganadobrijevic@gmail.com (D.D.); g.lye@ucl.ac.uk (G.J.L.)
4 Department of Biochemical Engineering, University College London, Bernard Katz Building, Gower Street, London WC1E 6BT, UK; jack.jeffries.12@ucl.ac.uk
5 Energy and Bioproducts Research Institute, Aston University, Birmingham B4 7ET, UK
6 Department of Chemical and Bioprocess Engineering, School of Engineering and Innovation, Aston University, Birmingham B4 7ET, UK
* Correspondence: j.ward@ucl.ac.uk (J.M.W.); m.cardenas-fernandez@aston.ac.uk (M.C.-F.); Tel.: +44-0121-204-3731 (M.C.-F.)

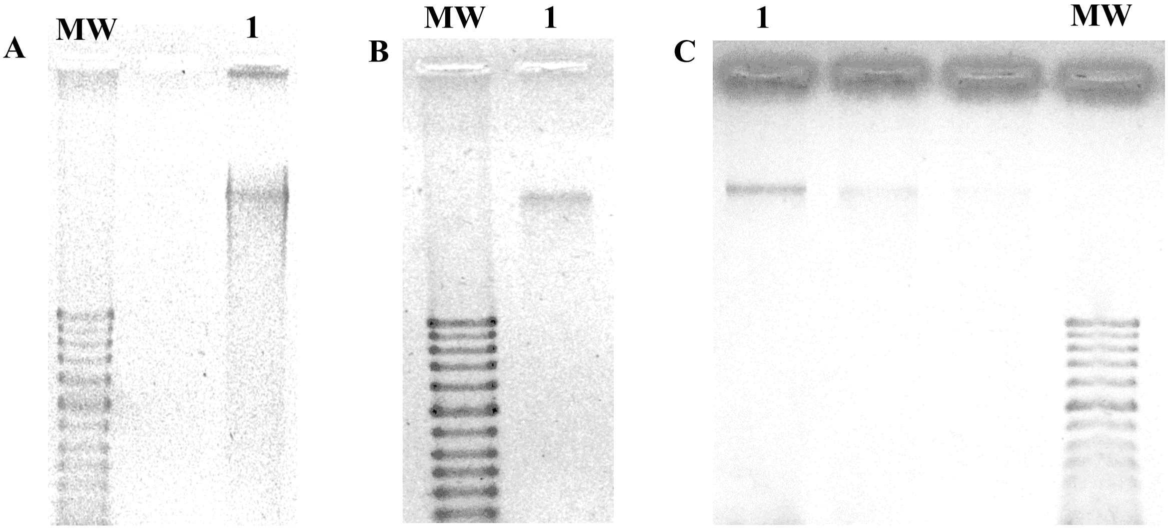

Figure S1. Agarose gel electrophoresis (1%) showing the extracted mDNA of soil samples from Peruvian salterns. (A) Maras3, (B) Maras6 and (C) Pilluana3. Lanes: 1, mDNA; and MW, molecular weight marker.

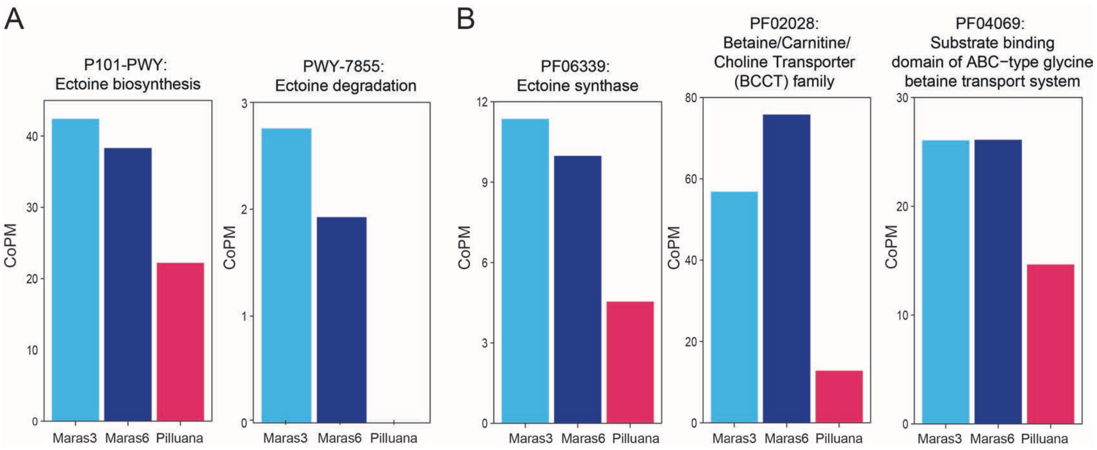

Figure S2. Functional analysis of ectoine and glycine betaine in shotgun metagenomic sequencing of Peruvian salterns soil samples using HUMAnN 3.0. Normalised abundance of **(a)** MetaCyc pathways involved in ectoine biosynthesis and degradation; and **(b)** Pfam domains involved in ectoine biosynthesis and glycine betaine transport.

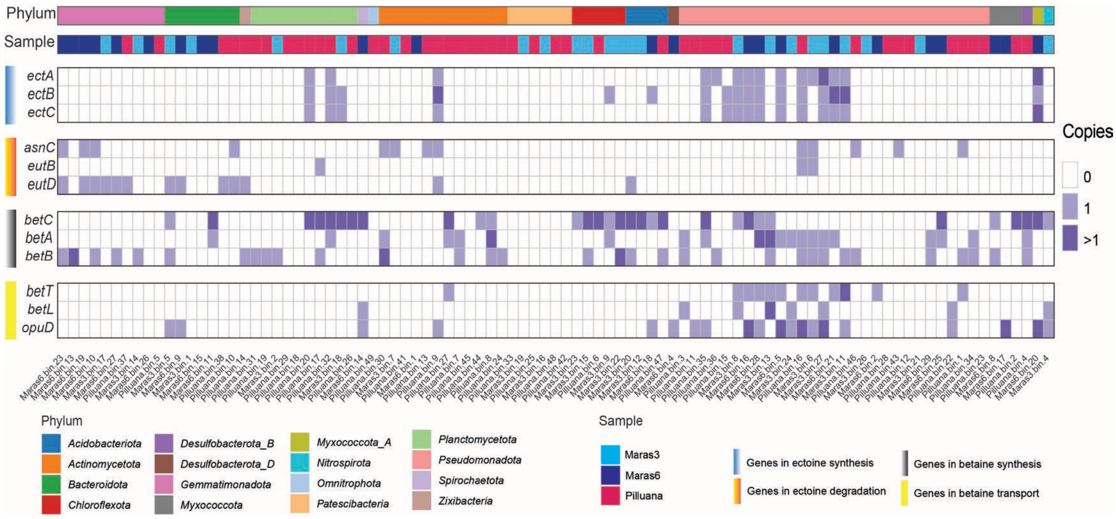

Figure S3. Heatmap showing the presence/absence of selected genes involved in ectoine synthesis and degradation, as well as glycine betaine synthesis and transport pathways in MAGs from Peruvian salterns samples. MAGs were functionally annotated using the 'annotate_bins' module in metaWRAP. MAGs (bins) are labelled with phylum (assigned by GTDB-Tk) and sample, as in Figure 7.

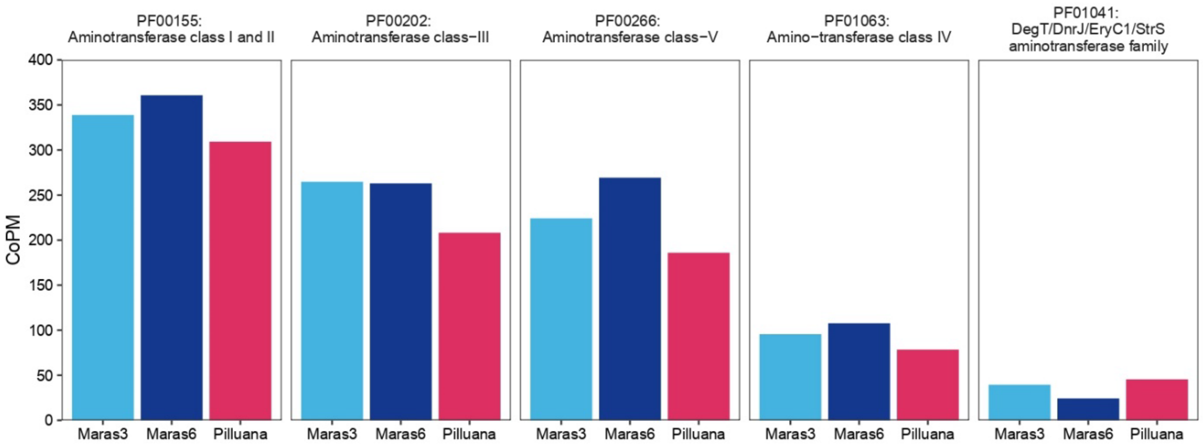

Figure S4. Distribution of metagenomic ATs in all site samples.

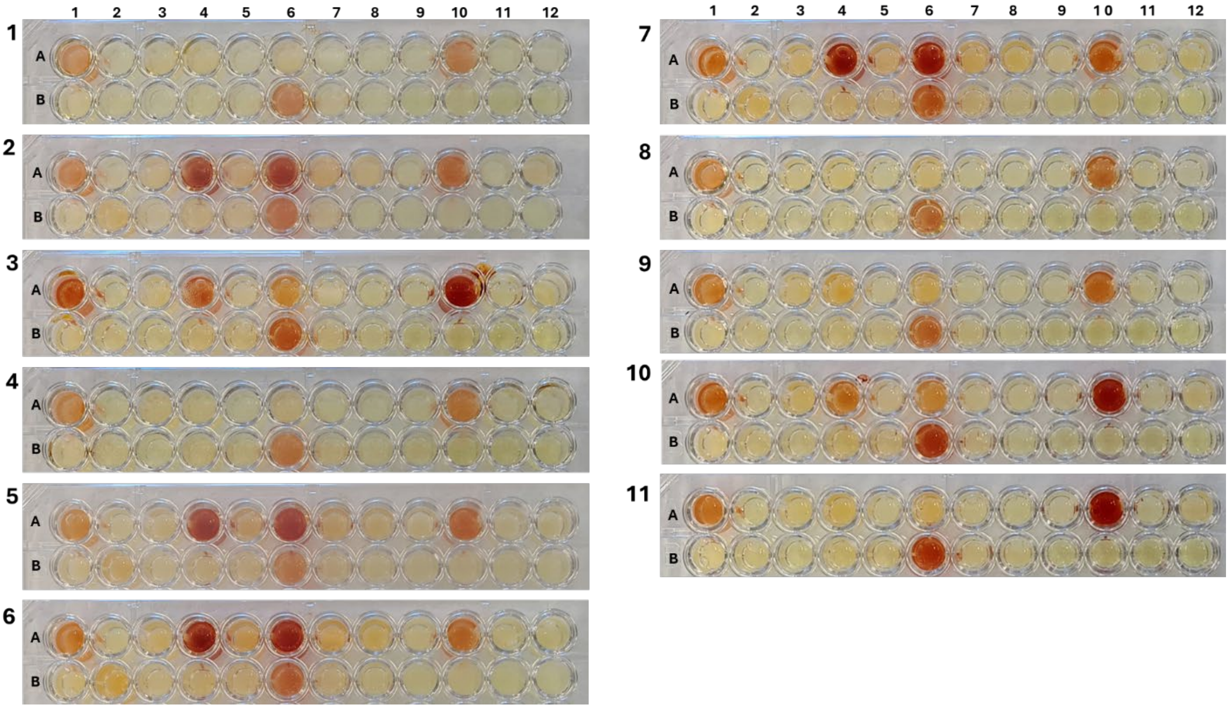

Figure S5. Aminotransferase substrate scoping assayed for the following aromatic and aliphatic substrates: (1) Acetophenone, (2) Benzaldehyde, (3) Pyruvate, (4) D-Xylose, (5) Furfural, (6) Methyl furfural, (7) Hydroxymethyl furfural, (8) 2-phenyl-2-butanone, (9) Acetoin, (10) 2-ketobutyric acid and (11) α-ketoglutaric acid. The reactions were carried out by mixing 20 μL of AT crude lysate with 180 μL of substrate mix containing 10 mM substrate, 25 mM 2-(4-nitrophenyl)ethan-1-amine as amino donor, and 0.2 mM PLP in buffer HEPES 50 mM pH 7. The reactions were carried out in sealed 96-well plates and incubated at 37 °C for 24 h. ATs from A1 to B8, pQR number as in Table S3. Negative control reactions in B9 (no enzyme) and B10 (no amino donor).

Table S1. Parameters of sample sites from Peruvian salterns.

Parameter	Maras3	Maras6	Pilluana
Location	13°18'10.8"S<br>72°09'15.5"W	13°18'09.0"S<br>72°09'16.2"W	6°46'04.3"S<br>76°17'24.4"W
Elevation (m.a.s.l)	3030	3030	200
Environmental temperature (°C)	-8 to 20	-8 to 20	17 to 40

Table S2. Gene sequences of the putative metagenomic aminotransferases class III isolated from Maras3. Plate position corresponds to the bioprospecting prospecting experiments (as shown in Figure 6 and Figure S4). pQR numbers correspond to the internal identifier codes.

Plate Position	pQR number	Gene sequence
A1	pQR3071	ATGATTTCCACAGGTGAAGCAATTAAGCTAGAAGATCAGTACGGTGCTCATAATTACCATCCTCTGCCTGTAGTTT<br>TAGCCAAAGGTGAAGGGGTTTATGTCTGGGATCCCGAAGGCAACAAATACTACGATTTCCTGTCGGCTTACTCG<br>GCCGTCAACCAGGGGCACTGCCATCCACGAATTATTAAAACGCTCAAAGAACAAGCCGAAAAATTAACTCTCGTT<br>TCCAGAGCTTTTTACAGTGATCAGCTGGGCAAGTATGAAAAATTCATACACAAAACCTTCGGCTACGATAAACTA<br>CTGCCGATGAACACCGGTGCTGAAGGTGTGGAAACAGCCATTAAACTTTGCCGAAAATGGTCATATGAAAAGAA<br>AAACCTAACGCCGGAAAAGGCCACGATTATTGTAGCTAAAGGAAATTTTCACGGACGCACAACCAGCATTATTTC<br>GTTTTCCAATGATCCGGTTGCCCGCAAAAACTTTGGTCCCTATACCCCGGGATTTGTCTCTATCCCCTATAACGATT<br>CGGATGCCCTTGAACAAGCACTTCAGGAAAAAGATGTAGCCGGATTTCTGGTTGAACCTATACAGGGAGAAGCC<br>GGCGTTGTTGTGCCCGATGAAGGCTATCTCCAGAAAGCATCTGAATTGTGCAAAAAGTACGATACCCTGTTTATT<br>GCGGATGAAATTCAGACAGGTATAGCCCGAACGGGAAAAATGCTGTGCGTAGACCACGAAAATGTGCGTCCCG<br>ATATCGTGATTCTCGGCAAAGCTCTCTCCGGCGGGGCGTATCCCGTATCCGCTATTTTAGCTGATGATGAAATCAT<br>GGAATCCATGCGTCCGGGCGTGCATGGTTCAACCTACGGTGGAAATCCGCTTGCCTGTGCCGTAGCGATGGATG<br>CGCTAAAAGTAGTTCAGGATGAAAATCTTGCTGAAAATGCCGACCGGTTAGGTAAGGTTTTTCGCGCTGAGATG<br>GAAAAACTCATCGAGGAAACAGATCTGGTAAAACTCGTGCGGGGAAAAGGACTGCTCAACGCCATAGTTATCAA<br>CGACTCAGCAGAAAGTGAAACGGCATGGAATATCTGCTTGAAACTCAAAGAGAACGGATTACTTGCCAAGCCTA<br>CTCATGGAAATATCATTCGCTTCGCACCTCCCCTGGTGATGACGGAATCTCAGCTCCGAAATTGCATTTCAATTAT<br>TAGGGATACGATTTATTCCTACGAGAAAGTACAAACGTAG

**A2**	**pQR3073**	ATGTCGATTTCAGAAAAAATTTCTTCAGAAGAAGCAATCCAGTTAGAAGATAGATATGGAGCGCATAATTATCAC<br>CCGCTGCCTGTAGTGCTCGAAAAGGGTGAAGGCGTATATGTTTGGGATGTGGAAGGAAAAAAATATTACGATTT<br>TCTTTCGGCTTATTCTGCAGTGAACCAGGGGCATTGTCATCCTAAGATCGTTGGGGCCATGCATGAGCAGGCTCA<br>AAAATTAACCCTTACTTCCAGAGCATTTCACAATAATATTTTGGGACAGTATGAGAAATTCGCTTCAGACTATTTT<br>GGTTTTGACAAGCTCTTACCCATGAATACGGGGGCAGAAGCTGTGGAAACCGCGATCAAGATCGCCAGAAAATG<br>GGCTTATGAGAAGAAAAATGTGAAGGAAACAGAAGCGCAGATCATTGTTTGTGAAAACAACTTCCACGGAAGA<br>ACCACTACAATTATCTCTTTTTCCAATGACGAGGAAGCCCGAAGGAATTTTGGACCATACACCCCGGGATTCCTGA<br>AGATCCCTTATAACGATACTGAAGCTCTTGAAGAAGCTTTAAAGAATAATGATAATATCGCCGGATTCCTTGTAG<br>AACCCATCCAGGGAGAGGCGGGAGTATATGTTCCTACTGAAGGATTCCTTGCCCGTGCGAAGGCCTTATGTGAG<br>GAACACAATGTGCTGTTCATTGCCGATGAAGTACAAACAGGGATCGCCCGTACCGGGAAACTGCTAGCCGTAGA<br>CCATGAAGAGGTGAAGCCTGACGTGCTTATTCTTGGAAAAGCCATTTCAGGTGGGGTTTATCCGGTGTCGGCAG<br>TTCTGGCGAATGACAGGATAATGAATGTAATACAGCCGGGTCAGCATGGATCTACCTTTGGTGGTAATCCTGTG<br>GCCTGTGCCGTGGCAATGGCCGCTTTAACGGTTATTAAAGACGAGGAGCTCGCCAAAAATGCCGAAGAGCTTGG<br>AAACCTCTTCCGTAGAAAATTAGATGATTATATCCGAACTTCGAATATCGTAAAGCTGGTAAGAGGTAAGGGGTT<br>GTTAAACGCCATCGTAATCAACGATTCTGAAGACAGTTCCACTGCATGGGATATTTGTATGAAGCTTAGAGACAA<br>TGGGCTGCTTGCCAAACCTACTCATGGAAATATCATTAGGTTCGCACCGCCGTTGGTGATGAATGAAGACCAGTT<br>GACTGACTGTGTAGAGATCATTACAAAAACTCTTGCGGAATTCGAAAAATAA
**A3**	**pQR3074**	ATGATTTCCACTATCGAAGCCATTGAACTCGAACATAAACTGGGAGCACATAATTACCATCCTCTTCCTGTAGTCT<br>TATCAAAAGGTGAGGGGATACACGTTTGGGATCCCGAGGGAAATAAGTATTATGATTTCTTATCAGCATATTCGG<br>CCGTAAATCAGGGGCACTGTCATCCACGGATTATTAACAAGCTCAAAGAGCAAGCCGAGAAATTGACGCTGGTT<br>TCACGGGCCTTTCACAGCGATCAGCTCGGTCAGTACGAAAAATATATGCACGATCTCTTTGGATACGATAAGCTG<br>TTACCTATGAACACCGGTGCCGAAGGTGTGGAAACAGCCATTAAACTTTGCCGAAAGTGGTCATACGAAAAGAA<br>AAACCTGACTCCCGAACAAGCTACGATCATCGTTGCCAAAGGGAATTTCCACGGGCGCACGACCAGCATTATTTC<br>ATTCTCTAATGATCCTCTTGCCCGCAAAAACTTTGGTCCCTACACTCCCGGATTTGTTTCTATTCCCTATGATGATTT<br>GGATGCACTGGAAGAAGCGCTTCAGAAGAAAGATGTTGCTGGATTTATGGTAGAGCCCATCCAGGGAGAAGCG<br>GGTGTTGTCGTACCAAGCGACGGCTATCTCACAAAAGCTGCAGAGTTATGCGAAAGATATGACACTCTTTTTATT<br>GCGGATGAAATCCAGACCGGCATTGCTCGCACGGGGAAAATGCTCTGCGTAGATCATGAAAATGTGCGCCCCGA<br>TATTGTGATTCTAGGGAAAGCCCTCTCCGGCGGTGCCTACCCCGTTTCGGCCGTTTTAGCAGATGATGAGGTTAT<br>GGAGTGCCTTCGTCCCGGTGAGCATGGATCTACATACGGCGGAAACCCTCTTGCCTGCGCCGTGGCGATGGAAG<br>CTCTCAAGGTGGTGGAGGATGAAAACCTCGCTGAAAATGCTGAACGACTTGGTAACATCTTCCGCGATGAGATG<br>AATAAACTAGTTGAGGAATCTGATTTAGTAAATCTCGTACGGGGCAAAGGACTATTAAACGCGATCGTCATCAAT<br>GATTCGGAAGAAAGCGACACAGCCTGGAATATCTGCCTGAAACTGAAAGAAAATGGGCTGCTGGCCAAACCCAC<br>TCACGGCAATATCATTCGATTTGCTCCTCCTCTGGTAATAACCGAAGAACAACTGCGCGACTGTATTTCAATTATT<br>AAGGATACCATTAAGACATACAAAGAAGTGATGGCATAG
**A4**	**pQR3075**	ATGTTGAGTATCAAAGATTCCTTTTACGAACACGTTGCCCAGACCAGCGATGCGCCCATGGGATTGGAAATCGAA<br>AGGGCAGAAGGGCCGTATATCTTTACAACCGATGGAAATAAATATGTAGATTTTATTTCCGGTATTGCGGTTAGC<br>AGTTTGGGGCACCGTCATCCCGAAGTAGTGAAAGCGGTAAAACGCCAGGTAGATCGCCATTTACATGTGATGGT<br>ATATGGCGAATTTATACAGGAGCCGCAGTCGAAATATGCTGAGCTGCTCACATCGCAACTGCCCGATTCGTTGGA<br>CCGCGTATACTTTGTAAACAGCGGTACCGAGGCCAATGAAGGTGCGCTGAAACTGGCCAAGAAACATACGGGTC<br>GCCATAAGCTTGTGGGATTCCATCATGGATATCATGGAGATACCCACGGCTCGTTAAGTGTGACCGGTAGGGAT<br>GTCTATCGTGATCCGTATTTGCCGCTGCTGCCTGATGTCCATTTTTTAGATTTCAATAGTTTTAATGGATTGGAAAC<br>TATTGACGAAGAAACGGCAGCCGTAATTATGGAGCCTATTCAGGGCGAAGGAGGCATCATCCCTGCAAAAAAAG<br>AGTGGCTACAGGCTATGCGCAAGCGCTGTAATGAAGTCGGGGCGCTCCTCATTTTTGATGAAATACAGACAGGA<br>TTTTACCGCACCGGTTCGCTGTTTGCTTTCCAGGGATACGGAGTGGAGCCTGATATTATGAGTTTGGCCAAGGCA<br>ATGGGCGGCGGTATGCCGATGGGGGCTTTTGTGTCTTCATCCGAAATATTTGAATCATTTAAACACGATCCGCCC<br>CTGAATCATGTGACTACCTTCGGCGGCCATCCGGTTTCGTGTGCCGCGGCCTATGCTACCTTGTCGGAATTACTTT<br>CTGGCGACTATGGAGCAAAGGCCCAAAAAGTTGAGCAAATCGTTCGAAAAGAGTTAACGGCTGAGGGAATCAC<br>AGAGATTCGCGGCGTTGGTGCGATGCTGGGAATGGAGCTGGAAGATCGTGAGTTAACCCAAAAGGTAGTGCAA<br>GAATGTATGGAGAAAGGTATCATATTAGGATGGACGCTTCATTCGGATACTCTGGTGCGACTGGCACCACCGCT<br>GATTATCAAAGAAAAATTGTTGCGATCAACTTTGCAGGTTATCAATAAGACCGTCAAAAAATATGGTTAA
**A5**	**pQR3076**	ATGTTGCCAAGTAAAGAATCTTTTTATGATCACGTAGCCCAAACCAGTGATGCCCCGATGGGACTGGAGGTCGAC<br>AGGGCAGAAGGATCGTCTATTTACACGACCGATGGCAAAGAATACGTGGATTTTATTTCCGGTATTGCCGTAAGC<br>AGTTTGGGTCACCGCCATCCCAAAGTTGTAGAAGCTGTTAAACGTCAAGTAGATCGTCACTTGCATGTGATGGTT<br>TACGGGGAATTTATACAGGAGCCTCAATCAGCGTATGCCGAATTGCTCACTTCACAATTACCGTCATCATTGGATC<br>GTGTGTACTTTGTGAACAGTGGAACCGAAGCCAACGAAGGCGCTCTGAAACTGGCCAAAAAGCATACCGGACGC<br>CATAAATTTGTAGCCTTTCATCACGGTTACCACGGGGATACACACGGTTCGCTCAGCGTTACTGGCCGCGATGTG<br>TACCGGGATCCATATTTGCCCCTGCTGCCGGATGTAGACTTTTTGGATTTTAATAGTTCTGAAGAATTGGAAACGA<br>TAGACGAAGAAACAGCCGCCGTCATCATGGAGCCGATCCAGGGGGAAGGAGGGATTATTCCGGCCCATAAAAA<br>GTGGCTAAAAACTGTGCGGCAGCGTTGTGACGAAGTGGGAGCACTACTTATTTTTGATGAAATACAGACCGGAT<br>TTTTTCGTACGGGATCACTTTTTGCTTTCAAATATTACGATGTAGTACCCGATATTCTGTGCCTTGCAAAGGCGAT<br>GGCCGGAGGCATGCCGATGGGAGCTTTTGTATCATCAAGCGAAATTTTCCAGACATTTATGCACGATCCGCCGCT<br>CAATCACGTCACCACATTTGGCGGACATCCGGTTTCGTGTGCTGCTGCGCATGCTACGCTAAAAGAGCTGCTGGA<br>CGGAAATTTTGAAGAAAAGGCGAAACAAATTGAAGCCATTGTCAGAGAGGAACTGACGGCGGGAGGTATTGTC<br>GAAATCCGGGGGCGGGGAGCTATGCTTGGAATGGAGTTGCGGAGCAAAGAAATCACACAAAAAGTAGTGGAA<br>GATTGTCTGGATAAAGGAATCATCCTGGGATGGACCCTCCATTCTGATACGTTGGTAAGACTTGCTCCTCCTCTAA<br>TTATTGATAAGGAATTGCTGCGGTCTACCTTGCAGGCAATAAATGAAGCGTAGCCAGGTTTAACTGA

**A6**	**pQR3077**	ATGAGTTCTAAAGAGCAGTTTTACAGCCATGTTGCCCAGACCAGTGAATCCCCAATGGGACTGGAAATAGACTAT<br>GCCGAAGGGCCCTTTATTTTTACTAAAGATGGGAACCGATATGTAGATTTCATTTCCGGAATAGCGGTCAGCAGT<br>CTTGGTCACCGCCATCCTGCCGTGGTTGATGCGGTCAAGAAGCAGGTAGATCGTCATCTACATGTAATGGTATAT<br>GGCGAGTTTATACAGGAACCGCAATCTCGGTATGCTGAGCTGCTTACCTCTCAGCTACCGTCCAAGCTCGACCGC<br>GTATACTTTGTCAATAGCGGTACGGAAGCTACCGAAGGTGCTTTAAAACTGGCAAAAAAGTATACCGGCAGGCA<br>CAAGTTTGTAGCCTTCAGGAACGGTTATCACGGCGACACTCAGGGCTCTTTGAGTGTTACGGGTAGAGATGTGT<br>ACCGTGATCCCTATCTGCCTTTGCTGCCGGACGTTCATTTTCTGGATTTTAACAGCAATGAAGGTCTGGATACCAT<br>TGACAATGATACAGCGGCTGTAATCATGGAACCTGTGCAGGGAGAGGGGGGCATAATTCCTGCAGATAGGGTG<br>TGGCTCAAAAAGGTGCGCGAAAAATGTACTGAAAAAGGCGCATTACTGATCTTTGACGAAATACAGACCGGCTT<br>TTACAGGACAGGCACTCTTTTCGCCTTTCAGGATTATGGGGTAGTTCCGGATATTATGTGTCTTGCAAAAGCGAT<br>GGGAGGCGGAATGCCTATGGGCGCATTCGTATCTTCGAGCCAAATATTCAAAACCTTTATGCATGATCCCCCACT<br>GAATCATGTCACTACCTTCGGTGGACATCCGGTATCTTGTGCCGCTGCCCACGCTACGCTTTCCGAGTTGCTAAAA<br>GGTGATTTTGCACAGAAAACCGAACAGATAGAAACGAAAGTGAGGCAGGTGCTAAAAGGAAACGGTATCATTG<br>AAATAAGAGGCAAGGGAGCCATGTTGGGCATGGAGCTGGAATCCAGAGAATTAACCCAGAAGGTGGTTCAGGA<br>GTGCCTGGATCAGGGTATTATCCTGGGATGGACGCTGCATTCCGAAACACTGGTCAGGCTAGCCCCGCCACTGA<br>TTATTGAGCCCAATGTCTTGGAAGACTCTTTACATACAATAATAGAGAGCATCAGGAAGTTTACAGGTCGGAATT<br>TAAATTAA
**A7**	**pQR3079**	ATGGATATAGAACAATCCTTTTACCGACATATTGCACAAACCAGCGACGAACCGATGGGACTGGTGATCGACCG<br>GGCGGAGGGGGCCTTTATTTTTACTGAGGATGGCACCCGCTATGTCGATTTTATCTCCGGCATCGCCGTAAGCAG<br>CCTGGGACACCGGCATCCGGCGGTCATTGAAGCCGTGCAACGGCAGTTAGACAAGCACCTGCACGTAATGGTGT<br>ATGGCGAATTCATCCAGGAACCGCAATCCCAATATGCCGAGCTGCTCACCGGTCAACTGCCCGAACTACTGGATC<br>GGGTGTATTTTGTAAACAGCGGCACCGAAGCCAACGAGGGAGCGCTTAAACTCGCCAAAAAGCATACCGGCCG<br>ACACAAATTCGTGGCTTTTCGGCACGGCTATCATGGTGATACCCACGGCTCGCTCAGCGTGACCGGGCGAGATG<br>TCTATCGTGATCCCTACCTTCCGTTGCTGCCCGATGTAGAGTTCCTGGAGTTCAACGATCCCAATGAGCTGGACGT<br>GATTGATAAAGATACAGCAGCTGTCATCATGGAGCCGGTCCAGGGAGAAGGCGGTATCATCCCCGCCCAAAAAG<br>AGTGGCTGCAGCAGGTCCGCCGGCGATGCGATGAAACCGGTGCACTCTTGATCTTCGACGAAATCCAGACCGGC<br>TTTTACCGCACGGGCTCGCTGTTTGCCTTCCAGTATTACGAGGTGGTGCCCGATATCCTGTGCCTGGCCAAGGCG<br>ATGGGCGGCGGCATGCCGATGGGCGCGTTTGTCTCATCCAGCGAAATATTTGAAGGCTTCAAGCACGATCCGCC<br>CCTCAACCACGTAACTACGTTTGGCGGGCATCCCGTAAGCTGTGCCGCGGCCCACGCCACACTTTCTGAACTGTT<br>AAATGGCGATTATGGAACGAAGGCGCGCTATATTGAGAACAAAGTTAAATCTACACTGAAACACCCGGCCATTG<br>CCGAAGTGCGCGGACGCGGTGCCATGCTGGGGATGGAGCTCCGGGATGCCGATCTGACCAAAAAGGTGGTGCA<br>ACGCTGTTTTGAAAAGGGCATCATCCTGGGATGGACACTTCACTCCGATACGCTGGTCCGCCTGGCGCCCCCGCT<br>CATCATCGAAGAATTGCTTTTAGAAGATACGCTGGAGACGATTCTGCGGAGCGTGGATCACTATGCATAA
**A8**	**pQR3080**	ATGAGTAATAAGGATTCATTTTATCAACATGTTGCACAAACCAGTGATGCCCCGATGGGGCTGGAGATTGACTAT<br>GCCAAAGGTCCATATTTGTACGACACCAATGGCAAGCAATACGTAGACTTTATCTCAGGCATAGCGGTCAGTAAT<br>CTCGGGCATCGCCACCCCAAAGTAGTGCAAGCTGTCAAAGAACAGCTTGACCGGCATATGCATGTCATGGTGTAT<br>GGAGAATTTATTCAAGAGCCACAGTCGGCCTATGCCGAGTTATTGACTTCTCAATTGCCTGACAAACTGGACCGT<br>GTATATTTTGTAAACAGCGGAACCGAAGCCAATGAAGGGGCACTGAAGCTGGCTAAAAAACACACCGATCGGCA<br>CAAATTTGTGGCTTTTAATCACGGCTATCACGGAGACACGCATGGATCGCTCAGCGTTACCGGTCGCGATGTGTA<br>TCGGGATCCCTATAAACCACTCCTGCCCGATGTGCATTTCTTGGATTTCAACCGTTTTGAGGCATTGGAGACAATC<br>GACCGAGAAACAGCGGCCGTTATTATGGAGCCGATACAGGGAGAAGGAGGGATCATCCCGGCAGAAAAAAAG<br>TGGCTGCAAGCGGTGCGCAAACGATGTGATGAGGTGGGAGCACTTCTCATTTTTGATGAGATTCAGACGGGATT<br>TTATCGCACGGGATCGCTGTTTGCCTTTGGGCAGTACGATGTAGTGCCGGATATTATGAGTCTTGCCAAAGCGAT<br>GGGGGGCGGCATGCCGATGGGAGCATTTGTATCCTCATCCGAAATATTCGAATCGTTTATGTATGATCCACCGCT<br>GAATCACGTGACCACATTCGGCGGCCATCCCGTATCATGTGCAGCAGCTCATGCTACTTTGCAAGAGCTTTTAGA<br>TGGGGATTTTGGAAGCAAAGCCCAACGTATTGAATCGGTCGTTCGGGAGGAATTATCCGCCGATGGCATTCAAG<br>AAGTTCGAGGTGTGGGTGCTATGCTGGGGATGGAATTAAAGGATAAAGATATCACCCAAAAGGTAGTAGAAGA<br>TTGCCTTGAAAAGGGGATAATTTTGGGGTGGACGCTCCATTCGGATACGTTAGTAAGATTAGCGCCCCCGTTGAT<br>TATCGAAGAAGAATTGTTGCGGAGAACCCTCCAAATTATTAACCAAAGTGTGTTAGAGTTTTCTTGA
**A9**	**pQR3081**	ATGAATTTTCAGCAGCTTACTGAACAGCATCACCTCCCTGTTTACAACCGATTTCCGATCACCTTAGTAAAAGGCA<br>AAGGGGCTCATGTGTGGGACAATAATGGCAACAAATATATCGATGCTTTAGCCGGCATTGCGGTCAATAGTTTG<br>GGGCATTGTCATCCCAATGTTGTAGAAGCAGTACGCAAGCAGGTTGGACAATTAATGCATATTTCCAACTTCTAC<br>TACAGCAAGCCACAGGCCAAATTGCTCGAGCTTTTATCACAAATTTCGAGCCTGGATAAAGGCTTTATATGTAAC<br>AGTGGTGGAGAAGCCATGGAAGCTTGTCTTAAAGCAGCACGCAAATACGGGCAAGCTCATCAGAAAGAAGGTT<br>CACTGTTGACCGTTAGCAATGCCTTTCACGGACGTACGGTGGGGACGATATCTATGGGTATGGATAAATATTCAA<br>AGGGCTATGATCCGCTATTAGGCGGTTTTCAGCAGATTCCGTTAAATGATGTAGAAGCATTGAAACAGAATTTCG<br>ATAGCGACACACTCGGTATTGTCATTGAAACCATTCAGGGCTCGGGCGGACTGCATGTGGCTTCGCAGGAATTTA<br>TGGATGCTATACAGGAGCTTTGCAATCATCACAATGCGCTTTTAATTGTGGATGAAGTACAAACCGGTATAGCAC<br>GCACCGGCAAAATGTTTGGTTTTGAGCATTACGGGGTAGAACCTGATATCATTGGGATGGCCAAGGCCATGGGC<br>GGAGGGTTCCCCATTGGAGCGATGGTCTGCTCCGATAATGTTGCGGAAACGATGAGTTATGGAGACCATGGCAG<br>CACGTATGGGGGAAATCCTTTGGCTTGTACAGCATCAATGGCAGCACTGAAGACCGTTATTGATGAAAACCTTAC<br>GAAAGTAGCTGCTGAGAAAGGTGATTTTCTCAAACATAAAATTAAAGACTTAAGCACCGACATTAGCGAAATTGT<br>CGATATTCGCGGACGGGGTTTGATGCTTGGTGTGGAGCTTTCGTTTAAAGGTCGCTCGGTTGTAGAAGAGATGA<br>TGCACCGGGGCGTTTTGTCCAATTGTACACAAGGTAATGTTATACGGTTAGTGCCGCCTTTGGTGATCAACGAAG<br>AAGATTTATCATTCTTAGCCGAAGTATTAATTTCAGCAATAAAAAAATCATCCCCATAA

**A10**	**pQR3082**	ATGCCGACCATTAACCTTGTCACTGAAATACCGGGACCCAGAAGCCTTGAACTGGTCGCCCGGCGCGAGGCAGC<br>AACAGCGCGCGGCGCGGCTAAGCTCACACAAATCGCCGTCGAAGAGGCCAGCGGCGCGGCGGTGCGCGATGTC<br>GATGGCAACACCCTGCTCGATTTTGCAGGGGGCATTGGCGTGCTGGCAGTCGGTCACCGTCCACCGCAGGTGGT<br>GGACGCGCTCAAGGTGCAGGCCGAAAAGCTGATCCACATGTGTGCCATCGTCAGCACCTATGAACCGTTCGTGC<br>GCGTAGCGGAACTGCTCAACGAGATCACGCCCGGCGACTTTGCAAAGAAGTCGGTGCTGCTCAACAGCGGCGC<br>GGAAGCGGTAGAGACAGCCGTCAAGATTTCCCGTGCCTACACCGGCAGACAGGCCATTATCGTCTTCGAAGGGG<br>CCTATCACGGGCGCACCAACATGACGCTGGCCATGACCAGCAAGTACGGATTGTTCAAGAAAGGGTTCGGTCCC<br>TTCGCGCCGGAAATTTACCGTCTGCCGTTCCCCAATCCTTACCGCACGCCGCCCGGCATGACCGAGGAAGAATAC<br>ATCGAATGGGCGGCCTGGCAGCTTGAAAATGCCTTCGTGTCACAGGTGGCCGGGGAGTCAGTGGCGGCCATTG<br>TCATCGAGCCGGTGCAGGGCGAGGGCGGTTTCATTCCCACGCCGCCACGTTTCATGCAGCGCATCCGTGAATTGT<br>GCGACGAGTACGGCATTGTCATGGTGGCGGATGAGATTCAGTGTGGGTTTGCGCGCACCGGCAGGCTGTTCGCC<br>ATTGAGCATTACGACATCGCACCTGACCTGATCACGACGGCCAAATCATTGGCGGCGGGTATGCCTCTGGCGGC<br>GGTGACCGGGCGGGCAGAGATCGTAGATGCGCCGCATCCGGGCGGGCTGGGTGGCACCTACAGCGGCAATCC<br>ACTGGCATGCGTGGCGGCCATTGAAGCCATCGACATGATGCGTCAACCGGCATTCCTGAACCGGGCGCAGGCCG<br>TCGGCGAGCGCATTCGCGGGCATCTGATGGCGATCAAAGAGGAAAACGATCTGGTCGGTGATGTGCGTGGATT<br>GGGCGCGATGCTGGCGGCGGAGATGGTGCTGGATCGTGAAACCAGGCAGCCCGCTGCCGAGCAAACCTCGCAG<br>ATCAACCAGGAGACGCTCAAGCGCGGCCTGATCACCATTCGCGCCGGGTTGTATAGCAACTGCGTGCGCTTCCT<br>GCCGCCGCTGAATATAACCGACGACGAGATCGACGAGGGCATGGCCGTTTTTGCCGAAGCGGTGCGCGTGGTG<br>AATATGGCGCGTAAGTCACTGGCCGAAGTTTAG
**A11**	**pQR3083**	ATGTCTAAATTTCATCCGAATATCTGGTATCCGTTTACCATTTTGAAAGAAGCCCCCAAGCCCCTCAAAGTGAAAA<br>GGGGAAAGGGACTTTGGCTGGAGCTCGAAGATGGTCGTAAAATCATGGATTGCATTTCCAGCTGGTGGGTAAAT<br>ACCTTTGGTCATGCTCATCCGAAAATTACACAGGCAATTGCTGAACAGTCAGATAAACTTGAGCATGTTGTTTTTG<br>CAAACTTTACGCATGATCCGGCTGAAAAGGCTGCAGAGATGATAGCTGATGCCTTGCCGGATTCGCTCAACCGG<br>GTTTTTTATTCCGATGATGGGTCAACTGCCGTGGAAGTAGCGATGAAGATGGCCTATCAATATTGGCGCAATAAA<br>GGACAAGAGCGCAAAAAATTTATCTGCTTTGAAGGAGCTTATCATGGCGACACGTTTGGTGCCATGTCGGCTGG<br>TGAACGTTCGATCTTTACAAATGTCTTCCAGGATCTGTTATTTGATGTGGAGTTTTTACCATATCCCGATACCTGGA<br>TTGGTGATACGACGGTTGGAGACCGGGAGGATAAAATTATTACGAAACTTGAAACAATGTTGAGTGAAGATCCG<br>GAAATGTATGCCGGAATTATGATTGAGCCACTGGTTCAGGGAGCAGGTGGTATGAAAATGTGCCGAGAAGATTT<br>CCTGCAAAAGTTACATTGGGTAAATCGCCAATTTGATACATTGCTTATTTTTGATGAAGTAATGACTGGATTTGGT<br>CGCACTGGTGATTGGTTTGCTTGTGATCGTGCGCAGGTAGAGCCGGACATAATTTGTATGGCCAAGGGACTTACT<br>GGAGGATTTTTGCCTCTCTCAGCGACTGTTGCCTCTGATGAAATATACGACACATTTTATAGCAGTGACCCCCAAA<br>AAACATTTTGGCATGGACACAGTTATACGGCCAATCCCCTGGGCTGTGTGGCTGCTATTACCGCTTGCGAGTTGA<br>TGGAGGAGTATAAGTCCGTGTACTCAAATATGGAGAAATGGCATTCAAATGAGCTTCACAAGCTGCGCGACCAT<br>CCCCGTTTAAAAAACCATCGGGTGAAAGGGACTATTGCTGCGGTAGATATCGATACCAATGGTCGTAATGTAGA<br>AGTTGATCCTTATACCAGCGAAGTTTTGAGAGATGAAGATGGGTACCTAAATTCAGTGGCTGATGATATAAAAAA<br>ACGATGTGTTGATTACGGGTTATTGTTGCGTCCACTAGGCAATGTGTTGTATTTGATGCCGCCCTATTGCACAACG<br>AGATCGCAACTTTCTGAAATGTATGAGGGAATTGCCAAATTGCTGGATGAATGA
**A12**	**pQR3084**	ATGTCCACCAATAAGAAGGTAACAGCTGAACAGGTTCATAAAATTTTAGGGAAGCACATGCTAACCGACGGCTA<br>CAATATTGTGCTCGATCTGGAAAAAAGTAAGAGTCCCTATATTTACGATGCCAAAAATGGCGATCGCTACCTGGA<br>CTTTTTTACCTTTTTTGCCTCCAATCCGCTGGGGATGAATCATCCCAGCCTTAACAATGAAGAGTTTAAAAGAAAA<br>TTGGGCAGAGTAGCCGTCAATAAACCTTCCAACTCTGATGTTTATACCGAGGAAATGGCCGAATTTGTAGATAAT<br>TTCGATCGGATCGGAATTCCGGATTACCTGCCGCACACATTCTTTATTTCGGGAGGAGCACTGGCCGTGGAGAAT<br>GCACTCAAAGTAGCCTTTGACTGGAAAGTGCAAAAGAATTTTCAAAAGGGATATCGTGAAGAAAAAGGACACAA<br>GGTCCTTCATCTCGACCAGGCATTTCACGGCCGGTCGGGCTACACTATGTCATTAACGAACACCGATCCCAAAAA<br>GGTAAAGTATTTTCCGAAATTTGACTGGCCGCGCATCAGCTCTCCGGCTATGACCTATCCAGCCACGGAAGAAAC<br>TATTCGAAACACGGTTAAACAAGAAGAACTGGCCATCGCCCAGGCGGAACAATATTTCGATAAATATAAAGATG<br>ACATAGCCTGTATCGTCCTGGAACCCATTCAAGCCGAGGGCGGCGACCGACATTTTCGACCGGAATTTCACCAGG<br>CACTCCGCGAGCTTGCAGATAAACACGAAGCGTTACTGATCTATGATGAAGTACAAACCGGAGTGGGATTAACC<br>GGCAAATTCTGGGCTCACGAACACTATGTAAAACCGGACATCCTGGCATTTGGAAAAAAAGCCCAGGTCTGTGG<br>AATATTAGCAAGCACCCGCATCGACGATATTGAAACGAATTGCTTTAACGTCTCATCAAGGATTAATTCCACATG<br>GGGCGGCAACCTAGTCGACATGGTTCGCTTCGGGCGCATTCTCCAGGTCATCGAAGATGAAAACCTCGTAGAAA<br>ACTCTGCAAAGGTCGGCGAATATCTCCTCGATAACATTGAGGAACTGGCTGAAGAATTTGAAATCGTTCAAAATC<br>CGCGTGGCAAGGGACTTTTCTGCGCTATTGATTTCCCAAGTACCGAGATCAGAAATGCCGTAATTAAGGAATGCC<br>TGAACAACAAGCTGATGATATTATCATGCGGACCAAGAACAATGCGTTTTCGTCCACCGCTGTCCATTTCAAACG<br>AACACATTGACGAAGGCATCGAAATTATCAAGGAATCTATTAAAACAGTTCTAGAAGACCATCAAAATACCAGAC<br>ATGCGGTATCTGTAAGTAATTAA

**B1**	**pQR3085**	ATGAACGCAGCACAAATTACAGATCAGTTCCATCTGGATGTTTATAACCGATTCCCTATCATCTTGGAAAAAGGTA<br>AGGGCGCCAGGGTCTGGGATACGGATGGCACCGAGTATATTGATGCCCTGGCAGGTATTGCCGTGAACAGTCTC<br>GGCCACTGCCATCCCAATGTAGTGAAAGTGATACAGGAGCAGGCCGCTCAACTCATGCATATTTCAAACTTTTAT<br>TACAGCAAACCCCAGGCAAAGCTGGTCAAACTGCTGGCAGAGATGAGCGGTTTCGACCGAGTATTTTTATGCAA<br>CAGCGGTGCTGAAGCCATGGAAGCTTGTCTGAAGGTGGCACGAAAATTCGGTCAGAAACATCAAAAACAAGGT<br>CCGCTTATTACGGTGAGCAATGCGTTTCACGGCCGAACCATGGCAACCATATCTATGGGCATGGATAAGTATGCC<br>AAAGGCTACGATCCCCTCTTAAACGGCTTTACAGAGATCGCTTTAAACGATCTTGATGCCTTGGAAGCTGAATTTA<br>ACGAAAATACACTAGGCGTGGTACTGGAAACCATCCAGGGCTCCGGCGGACTTCACGTTGCAAGCAAAGCATTC<br>ATGGAAAAGGTGCAGGCTCTATGCCAAAAGTTTAATGCACTCTATATAGTTGATGAAGTCCAGACCGGTATGGG<br>CAGAACGGGAAAAATGTTCGGATTTGAGCATTATAATGTAGAACCTGACATCATAGCCACAGCTAAGGCTATGG<br>GCGGCGGTTTCCCTATTGGAGCCATGCTCTGCCGCCGGGAAGTGGCAGAGGTAATGAAACATGGCGATCACGG<br>CAGCACCTATGGAGGTAACCCGCTGGGCTGTGCTGCGGCCTATGCAGCTATTACTACAATTATCGATGAGAAACT<br>CCCTGAAGAGTCCCGGAAAAAAGGAACATACCTCAGGGATAAACTAACCGAAGCTTCCGAAAGTATGGCATCTA<br>TTGTCGATATTCGCGGGCGTGGGCTCATGATCGGGGTGGAACTCAGCTTCCCGGGGCGTCAGGTTGTGGAAGAA<br>ATGTTAAAAAAAGGTGTATTGTCGAATTGTACTCATGGGAATGTTATGCGACTGGTCCCCCCTTTAATTACTACCC<br>GCAAGGAACTTGATACCATTGCAGAGGTGTTGATTGAATCTGTTACCCTGGTCCAATAA
**B2**	**pQR3086**	ATGTCCACGAATAACACAGTCGCTCCCGCACAAGTACACAACATTTTACAAAAACATATTTTAACCGACGGCTATG<br>ATATTGTGCTCGATCTCGAGAAGAGTGAAGGTACCTATCTGTATGATGCCAAAACGGGGGAACGATATCTGGAT<br>TTCTTTACTTTTTTCGCCTCCAATCCACTGGGAATGAATCATCCACAACTGGCAAATGAAGAGTTCCGCAATAAAA<br>TTGGGAAAGTGGCGATCAACAAACCGTCAAACTCGGATGTGTATACCGAGGAAATGGCCGAATTTGTAGATAAT<br>TTTGATCGCGTCGGCATCCCCGATTACCTGCCCTACTCCTTCTTTATTTCTGGAGGGGCACTGGCCGTTGAGAACG<br>CCCTGAAAGTTGCCTTCGACTGGAAAGTGCAAAAGAATTTCCAAAAGGGCTATCGCCAGGAAAAGGGCCATAAA<br>GTTCTCCACCTCGAACAGGCTTTTCACGGGCGCACCGGGTATACGATGTCACTGACCAATACCGATCCCAAAAAA<br>GTGAAGTATTTCCCAAAATTTGACTGGCCGCGAATTATTTCACCGGCCATGAAGTATCCCGCTACGGACGAACAT<br>ATTCAAGAAACAATAGCAGATGAAGAGCGGGCCATTGCCCAGGCTAAGCGCTACTTTGAAATGTATAAAGATGA<br>AATCGCATGTATCATTCTTGAGCCTATTCAGGGAGAAGGCGGCGATCGTCATTTTCGCAAGGAATTTCACGAAGC<br>ACTAAGGAATTTGGCCGATAAACACGAAGCGCTTCTCATTTATGATGAGGTACAAACCGGTGTTGGACTAACCG<br>GAAAATTCTGGGCTCACGAACACTATGTAAAACCCGATATTCTGGCGTTTGGCAAAAAGGCGCAAGTATGTGGA<br>ATTCTTGCCAGCGAGCGCGTTGATGATATCGAAACCAACTGTTTTCATGTTTCCTCGCGAATTAATTCAACCTGGG<br>GCGGCAACTTGGTTGACATGGTTCGGTTCGGCCGCATTCTCCAGGTGATTGAAGAAGAGAATCTTGTTGAAAAT<br>GCCGCTACTGTTGGGAATTACCTGCAGGATAAAATCCAGAACCTTTCCGAAAAATTTGAACACGTCACTAATCCC<br>CGTGGTAAAGGACTTTTCTGTGCCGTTGATTTCCCCAATACTCATGCCCGTGATTCGGTTATCAAGGAGTGCTTCA<br>ACAATAACTTAATGATTCTGTCATGCGGAGAGCGAACCATGCGTTTCCGTCCGCCGCTTACAGTTAACAAACAGC<br>AGATCGAAGAAGGAATCGATATTATAGAAAAGTCCGTCAAATCGGCGATGGACAAATGCCCGGCACTGAAGAA<br>CACCGATAATTGA
**B3**	**pQR3087**	ATGCTTTATCACAAAACCTGGCATTTTNNNNNNNNNNCCGATCAGTTTCACCTAGACGTTTATAACCGCTTCTCT<br>ATTACATTGGCTAAGGGAGAAGGCGCCAGGGTCTGGGATACCGATGGAGTGGAATATATCGATGCCCTGGCGG<br>GTATTGCCGTTAACAGCCTCGGGCACTGCCACCCTGATGTTGTAAAAGCGATACAGGACCAGGCTGCACTGCTCA<br>TGCACATATCTAACTTCTACTACAGTGAACCCCAGGCAAAGCTGGTTAAACTGCTTGCAGAAATAAGCGGGTTCG<br>AGCGCATCTTTTTGTGCAACAGCGGGGCTGAAGCTATGGAGGCCTGCCTGAAAGCAGCTCGAAAATACGGACAG<br>AAACACCGAAAGACAGGTCCGCTGATTACAGTAAGCAACGCATTTCACGGCCGGACCATGGCAACCATATCAAT<br>GGGCATGGATAAGTATGCCAAAGGTTACGATCCGCTTTTAAGCGGCTTTGATGAAGTACCCTTAAATGATCTTGA<br>TGCCCTTGATGCTGCCTTCAATGGTAATACGCTAGGCGTGGTACTGGAAACTATTCAGGGCTCCGGGGGACTTCA<br>TGTGGCCAACGAAGCATTCATGAAAAAAGTGCAGTCTCTTTGCCGAAAACACGGGGCACTTTTTATTATTGATGA<br>GGTTCAGACCGGGATGGGCAGAACCGGAAAAATGTTCGGCTATGAGCACTATAGTGTGGAACCGGACATTATA<br>GCCGTAGCCAAGGCCATGGGGGGCGGTTTCCCTATAGGGGCTATGCTCTGCCGCCAGAAGGTGGCGGAGGTTA<br>TGAAACACGGTGATCACGGCAGTACTTATGGTGGAAACCCGCTAGGCTGTGCTGCAGCCCATGCCGCCATTACT<br>GCCATCATGGTAGAGAAGCTGCCGGAAGTGGCTCTGCAAAAAGGCGTCTACCTAATGGATAAACTGAAAGAAG<br>CTGCCAATAGTTTGGAATCCGTTGTCGATATTCGCGGAAGAGGCCTTATGATCGGGGTGGAATTGAGTTTTCCGG<br>GGAGGCAGGTTGTTGAAGAGATGCTGGAGCGAGGCATCTTATCGAACTGCACTCATGGAAATGTCATGCGTCTG<br>GTTCCTCCCCTAGTTGCCACCCGAAAGGAACTGGATACCATCGCTGAGGTGTTGGTAGAATCAATCAAATCTGCC<br>CAATAA

**B4**	**pQR3088**	ATGAAAATTTTCGACGAGATCGAATCCGAAGTGCAAAGCTACGCCCGCTCTTTCCCCAAGCTTTTTGAGCGAGCC<br>AAGGGCGAGTTCATGTACGATAAGGAAGGTAACGAGTATCTGGATTTCCTGGCGGGGGCCGGCACGCTCAATT<br>ACGGCCACAACAACGACCATTTCAAGAGCGCGCTGCTGGAGTACGTCCAGAACGACGGCATCACCCATGGTCTG<br>GATCTGCACACCACCGCCAAGGAAGATTTTCTCAACGCCCTGAACGACAAGATTCTCCGGCCCCGCGGCCTGGAG<br>TACATGGTGCAGTTCACCGGCCCCACTGGCACCAATGCCGTCGAGGCGGCGGTCAAGGTGGCCCGCAATGTCAC<br>CGGCCGGGAAAACATTGTCTCCTTCACCAACGGCTTCCATGGCGTGACGTTGGGGTCGCTGGCGATCACCGGCA<br>ATTCCCACCACCGGGGCGCTGCCGGCATCACCATGGGTGGCGTTACCCGCATGCCCTATGACGGTTATCTGGGG<br>ACGGATATTGATACCACCGCCTATCTGGACAAGGTGTTGAGTGATTCATCAAGCGGTGTCGATCTGCCCGCCGCT<br>GTGATTGTCGAGACCGTCCAGGGCGAGGGGGGCATCAACGCCGCCAGCACCGAATGGCTGCGCAATCTGTCCG<br>CCGTGTGCAAGAAGCACGAGGTACTGCTGATTGTCGACGATATTCAGGCCGGCTGCGGCCGCACCGGCACGTTC<br>TTCAGCTTTGAAGAGGCGGATATCCGCCCGGATATTGTCACCCTGTCCAAGTCCCTTAGCGGCTACGGCCTGCCT<br>TTCGCGGTCGTGCTGCTGCGCCCCGAGCTGGACCAGTGGCGTCCCGGCGAGCACAACGGCACCTTCCGCGGCAA<br>TAACATGGCTTTCGTTACCGCCAAGGCGGCCATCGATCATTATTGGTCGGACGACAGTTTCGCCAAAGAAGTGCG<br>GCGCAAGGGGGACTATATTCGCCAGCGCCTGAGCGCAATTGTGGAGCAGTATGGCGACGGCAACATGACCGCC<br>CGAGGCCGCGGCATGTTCCAGGGGCTGAACTGCGTCAGCGGCGACCTGGCGGACAAGATCACCAGCCTGGCGT<br>TCAAGAAGGGTCTGATTATCGAAACTAGCGGTGCCGATGACCACGTGGTCAAGACCCTGTGCAGCCTGACCATC<br>AGTGACGAGAACCTGAAGAAAGGGCTGGATATCGTCGAGGCGAGTGTCCGTGAAGTGTTGCGCGGCGACACCG<br>AAGTGCCCGCGGAAGAGGACTTTTTTTCCGACGACTGGGAAGCGCCCATTAAGCTGATCGTCAATAAATAA
**B5**	**pQR3089**	ATGAATTACCAGCAGCTTACGGAAAAGTATCACCTCCCTGTTTACAATCGATTTCCCATTACGCTAGTCAAAGGCG<br>AAGGGGCGCACCTTTGGGATGATAACGGCAACAAATATCTCGATGCATTAGCTGGCATTGCCGTAAACAGTCTG<br>GGACACTGTCACCCAAAAGTTGTGGAAGCTGTACAAGATCAAGTAGGACAGCTGATGCATATCTCCAACTTTTAT<br>TACAGCAAACCTCAGGCAAAACTGTTGGAGTTGCTCACTGAATTATCGGGCCTCGATAACGGTTTTCTATGCAAC<br>AGCGGTGGCGAAGGAATGGAAGCCTGCCTGAAGGTTGCCCGAAAATACGGACAAGCGCACGAGAAAACAGGT<br>CCGCTGATTACCGTAAGCAACGCTTTTCACGGCCGGACGATGGCAACGATCTCGATGGGTATGGATAAATATTCA<br>AAGGGGTATGATCCGCTCTTGCCGGGTTTTCAGCAGGTATCCATGAATGACATTGACGCTCTGAAATCTAATTTC<br>GACAATCAGACACTTGGGATTGTCCTTGAGACGATCCAGGGATCCGGTGGACTTCACGTCGCCTCACAAAAATTT<br>ATGGATGCCATCAGAGAACTTTGTCAATCAAATAATGCGTTGCTCATTATCGATGAAGTACAGACCGGAATTGGC<br>CGTACGGGTAAAATGTTTAGCTATGAACATTACGGTGTTGAACCCGACATCATTGCCATTGCAAAAGCGATGGGC<br>GGAGGATTCCCAATCGGCGGTATGCTCTGCAAAAAGAAGATAGCTAATGTAATGAGTTTTGGTGATCACGGAAG<br>CACATATGGAGGAAATCCTCTGGCTTGCCGCGCCTCAATTGCTGCTCTTAACGCCATTCGTGATGAAGGACTAGT<br>CGATCAGTCCAGAGAAAAAGGACAATTTCTGAAGTTTAAAATCAAAGAATTAGCCGAAGATGTCCCTCACATTAT<br>TGATATCAGGGGCAAAGGGTTGATGATTGGCGTGGAGCTTTCCTTTGAAGGACGAGCGGTCATCGAGGAAATG<br>ATGCGGCAAGGTGTATTGTCGAACTGCACGCAGGGCCACGTTATCCGCCTGGTACCGCCTTTGGTGACCAGCAA<br>AGAAGATTTGTCAAAACTAGCCTCCGTGTTGATAACATCTATTAAAAAAACTGCACCTGCAGAGAAAACAAATGG<br>CTGA
**B6**	**pQR3090**	ATGGCCACAGAAGCGCTGATGAATACCTACGGAGTGCGTCAACTGACAATGGCTCGCGGCGAGGGCTGTTACCT<br>TTGGGACGATCAGGGCAGGAAGTATCTGGATGCCCTATCCGGCATCGCTGTGTGCGGTCTGGGCCACGCCCACC<br>CGGCGGTCACCCGTGCCATCAGCGAACAGGCCGGCAGGCTGGTGCACTGCTCCAACTTTTACAATATCCCGGTG<br>CAGGAGGAACTGGCGCAGAGGCTGCATCAGTTATCGGGGATGACCAACTGCTTTTTCAGCAATTCCGGCGCCGA<br>GGCCAATGAAGCGGCCATCAAGCTGGCCCGACTCTATGGCAATAGCCGCGGCATCAAGTCTCCCACGGTCATCG<br>TAATGGAAAAGGCGTTTCACGGCCGCACCATGGCCACTCTCACCGCCACCGGCAACCGCAAGGTCCAGGCGGGC<br>TTCGAGCCCCTGCTGAAGGGTTTCACCCGCGCACCCTACAATGATGTCGCCGCCCTGGAAACCATTGCCGAAAAC<br>AACCCCGACGTGGTGGCCGTGCTGGCGGAACCCATTCAGGGCGAGGGGGGGCTCAATATTCCCGCCGATGATT<br>ATCTGCGGGAATTGCAGCGTGTCTGCCGCAGGAATGAGTGGCTGCTGATGCTGGATGAAGTGCAGACCGGCAA<br>CGGCAGAACCGGCAGCTTCTTCCACTATCAGCAAAGCGGCATTCTGCCGGACGTAGTCACCACCGCCAAGGGGC<br>TGGGTAACGGCTTTCCCATTGGCGCCTGCCTGGCTTCGGGTGAAGCGGCCACTTTACTCAAGCCCGGCAGCCACG<br>GTTCCACCTTTGGCGGCAACCCGGTCGCCTGCGCCGCCGCCCTGGCCACCCTCGACGTGATTGACAACGACAACC<br>TGACAGAGCGCGCCGCCGAACTCGGCCAGCGCCTGCTGACAGGCTTTGCCGACAGGCTTCAGGGCTGTGCGGG<br>CATCGTGGGTATCCGCGGTCGCGGCCTGATGCTGGGCATCGAGCTGGACCGCCCCTGCGGCGAACTGGTGGGC<br>CGGGCCGCCGGGGCGGGCCTGCTGATCAACGTCACCGCCGAGAAGGTGGTACGGCTGCTGCCGCCGCTGATCC<br>TGAGCGACGGCGAGGCGGACGAGCTGATGGACACCCTCGCCCGTCTTATCCGCGATTTTCTCGCCACCTGA
**B7**	**pQR3091**	ATGAACAGAGAACCCGTCAGCCGCGAATTATTCGATGATGTAATGGTCCCCAATTACGCACCGGGGTCTGTGATT<br>CCCGTGAAAGGGGAGGGCTCCCACGTATGGGATCAGGAGGGAAAGGAGTACATCGATCTGGCGGGTGGTATT<br>GCGGTGACCTGTCTCGGGCATTCCCACCCCGGCCTGGTCAGCGCCCTGACGGAGCAGGCCGAGAAAATCTGGCA<br>TCTGTCGAATGTAATGACCAACGAACCGGCACTACGGCTGGCGAAAGCCCTGTGTGACCATACCTTTGCCGAGC<br>GGGTTTTCTTCGCCAACTCCGGAGGGGAGGCCAATGAAGCTGCCTTCAAGCTGGCCCGCCGGTACGCCTGGGAG<br>CATTTCGGCCCGGAGAAGAACGAGATCATTTCCTTCAAGAGCTCGTTTCATGGCCGTACATTGTTTACGGTCAGC<br>GTCGGCGGCCAGCCCAAATACCTCGAGGGCTTTGAACCGGCACCAGGAGGCATTCACCATGCCGACTTCAACGA<br>TCTGGAGTCGGTCAGGAAGCTGATATCCAAGGAAAAAACCTGCGCCGTCGTGGTAGAGCCGATCCAGGGCGAA<br>GGCGGGGTTATGCCCGCTGACCCTGAATTCCTGAAGGGCTTGCGCCAGCTCTGTGACGACAACGATGCTCTGCT<br>GGTATTCGACGAAGTCCAGTCTGGCGTCGGCCGCACGGGACATCTTTATGCTTATCAGATGTACGGCGTAACGCC<br>GGATATCCTGTCAACAGCGAAGGGGCTTGGCGGTGGCTTCCCGGTGGCCGCTATGCTGACCACGGAAAAAGTC<br>GCGAAAAGCCTGGGAATTGGCACCCACGGTAGTACTTACGGCGGCAACGCTCTTGCTTGCGCGGTTGCTCAACG<br>GGTTATCGATACGGTCAGCCAGCCGGACATTCTCAAGGGCGTTGCGGCTCGCTCCGAGCGGCTGCGCAAGGGCA<br>TGATGGACATCGGCGAGCGCTACGGCATTTTCAGTGAGGTTCGTGGCTCTGGCCTGCTTCTCGGTTGCGTCCTGA<br>CGGACGAATGGAAGGGCAAGGCAAAAGAATTTCTCAACGCCGGTCTGAATGAAGGCGTGATGGTGTTGATTGC<br>TGGCCCCAACGTGGTGAGGCTGGCCCCGTCACTGATCATTCCCGAGTCGGATCTTGATGAAGCGCTGGTGCGGT<br>TTGAAGCTGGTGTAAAAAAGGTTTGCGGAAAATAA

**B8**	**pQR3092**	ATGTCATTTGCACACCCCACCGGCAACGTATTCTATCGCAAGTTAGCTCACCAACGCCCGATGATTTCACATGGGC<br>ATGGTGTCTATCTGTTCGATAAGGACGGGAAGCAATATCTGGACGGCTCCGGCGGCCCTCTCGTCGTCAATGTCG<br>GTCACGGCCGTTCGGAAATCGTACAGGCCATGGCTGAGCAGGCACAGTCTGCAGCCTACGTCCACGCCATCATG<br>TTCACCAGCGAACCGCTCGAAACCTACGCGCGCGAGCTGGCGACGACCCTGCCACTACCTGATCCGCGCCTCTTT<br>TTCCTCAGCAGTGGCTCCGAAGTCATCGAAGGCGCCATCAAATTAGCGCGACAAATCCAGATGGCGCGCGGCCA<br>TCCGGATCGCCATCTCATCATCTCCCGCTGGCAAAGCTATCACGGCACAACGCTGGGAGCGTTAAGCGTCAGCG<br>GTCGCCGGGGATTGCGCGCCCCTTATCTCAACATGCTGCAAGATATGCCGCACATTGCGCCTCCCTACCCTTATCG<br>CAATTCAGCCAGCGGCGAAGAGCTGGCATCCCACCTGGAAGAGGCTATTGAGACCCACGGACCTGAAAACGTCG<br>CCGCCTTCATCGCCGAACCTGTCAGCGGCGCCAGCCTGGGCGCCGTGGTGCCCCCACCCGATTACTGGCCGGCC<br>ATCCGTGAAATATGTGACCGTTACGACGTACTGCTCATCGCTGACGAAGTGTTGTCAGGAATGGGGCGTTGTGG<br>TCACTGGTGGGCGATTAGCCAGTGGGACATCGCACCTGACATCCTTGTTGCGTCAAAGGGCATTGCCGGCGGTT<br>ACTTCCCACTCGGTGCCCTTGCCACCAGTGGCTCAAATGTTGACCTGATTCAAGAGAAGCTCGGAGATTTTAATC<br>ATGGTGGCACCTTCAGCCACCACGCCGTTGGCGCAGCAGCAGGGCTGGCTACCTTGCGCATCATGCACGATGAA<br>AAACTGGTGCTCCGTGCGGCGACTGTAGGAGAAACAATAGGCGCCCGATTGCGCGACGCACTCGCTGACCATCC<br>GCACGTTGGCGACATTCGCGGTCAGGGCTTCTTCTGGGCGCTGGAGCTGGTTGCAGACCGCCAGAGTAAACTGC<br>CGTTTCCCGCCGCTGACCAGGTCGCCTGGCGCGTCTGGCAACGCGCTTTCGACCTTGGCCTCATTGTCTACTACTC<br>GCTGGGCTGCGCCGATGGCCGTAACGGCGACCTGCTCATGTTAGGTCCGCCGCTCATCACTACCGACGCCCAGCT<br>GGATGAGATAGTGGCACTGCTGGCCGCCGCGGTGGAGCAAGAGCTTCCGGGGCAGTCGCGAATATAA

Table S3. Alpha diversity metrics for the Peruvian salterns samples.

Sample	Shannon	Simpson	Berger-Parker
Maras3	8.1497	0.9991	0.01731
Maras6	7.9622	0.9990	0.01168
Pilluana	7.7984	0.9968	0.04269

Table S4. Archaeal MAGs recovered from Peruvian salterns samples.

Bin	Sample	Genus	Completeness (%)	Contamination (%)	Genome size (Mb)	GC content (%)
Maras3.bin.25	Maras3	*Halorussus*	53.01	7.87	2.12	65.6
Maras6.bin.22	Maras6	*Halorubellus*	82.61	1.55	2.60	66.0
Pilluana.bin.40	Pilluana	*Nitrosotenuis*	61.48	1.94	1.23	41.9
